# STIM1 translocation to the nucleus protects cells from DNA damage

**DOI:** 10.1093/nar/gkae001

**Published:** 2024-01-15

**Authors:** Irene Sanchez-Lopez, Yolanda Orantos-Aguilera, Eulalia Pozo-Guisado, Alberto Alvarez-Barrientos, Sergio Lilla, Sara Zanivan, Christophe Lachaud, Francisco Javier Martin-Romero

**Affiliations:** Department of Biochemistry and Molecular Biology, School of Life Sciences, Universidad de Extremadura, Badajoz 06006, Spain; Institute of Molecular Pathology Biomarkers, Universidad de Extremadura, Badajoz 06006, Spain; Department of Biochemistry and Molecular Biology, School of Life Sciences, Universidad de Extremadura, Badajoz 06006, Spain; Institute of Molecular Pathology Biomarkers, Universidad de Extremadura, Badajoz 06006, Spain; Institute of Molecular Pathology Biomarkers, Universidad de Extremadura, Badajoz 06006, Spain; Department of Cell Biology, School of Medicine, Universidad de Extremadura, Badajoz 06006, Spain; Bioscience Applied Techniques Facility, Universidad de Extremadura, Badajoz 06006, Spain; CRUK Scotland Institute, Switchback Road, Glasgow G61 1BD, UK; CRUK Scotland Institute, Switchback Road, Glasgow G61 1BD, UK; School of Cancer Sciences, University of Glasgow, Switchback Road, Glasgow G61 1QH, UK; Cancer Research Centre of Marseille, Aix-Marseille Univ, Inserm, CNRS, Institut Paoli Calmettes, CRCM, Marseille, France; OPALE Carnot Institute, Paris, France; Department of Biochemistry and Molecular Biology, School of Life Sciences, Universidad de Extremadura, Badajoz 06006, Spain; Institute of Molecular Pathology Biomarkers, Universidad de Extremadura, Badajoz 06006, Spain

## Abstract

DNA damage represents a challenge for cells, as this damage must be eliminated to preserve cell viability and the transmission of genetic information. To reduce or eliminate unscheduled chemical modifications in genomic DNA, an extensive signaling network, known as the DNA damage response (DDR) pathway, ensures this repair. In this work, and by means of a proteomic analysis aimed at studying the STIM1 protein interactome, we have found that STIM1 is closely related to the protection from endogenous DNA damage, replicative stress, as well as to the response to interstrand crosslinks (ICLs). Here we show that STIM1 has a nuclear localization signal that mediates its translocation to the nucleus, and that this translocation and the association of STIM1 to chromatin increases in response to mitomycin-C (MMC), an ICL-inducing agent. Consequently, STIM1-deficient cell lines show higher levels of basal DNA damage, replicative stress, and increased sensitivity to MMC. We show that STIM1 normalizes FANCD2 protein levels in the nucleus, which explains the increased sensitivity of STIM1-KO cells to MMC. This study not only unveils a previously unknown nuclear function for the endoplasmic reticulum protein STIM1 but also expands our understanding of the genes involved in DNA repair.

## Graphical Abstract

**Graphical Abstract:**
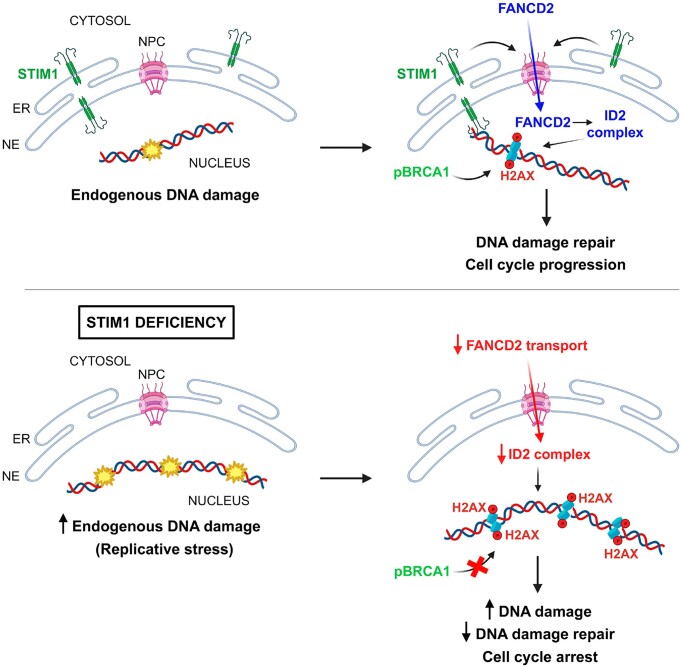

## Introduction

STIM1 is an endoplasmic reticulum (ER)-resident protein whose function is to regulate plasma membrane Ca^2+^ channels (1,2). Mediated by a Ca^2+^-binding domain oriented toward the ER lumen, STIM1 acts as an intraluminal Ca^2+^ sensor. When the Ca^2+^ concentration decreases below a certain threshold, STIM1 undergoes a conformational change, resulting in clustering and relocalization toward regions of ER and plasma membrane (PM) junctions (3–5). In these regions, STIM1 regulates the opening of plasma membrane Ca^2+^ channels by direct interaction of the cytoplasmic domain of STIM1 with the Ca^2+^ channels (6). This domain is known as STIM1-ORAI1 activating domain (7), because one of the channels regulated by STIM1 is ORAI1 (8). Consequently, Ca^2+^ influx through STIM1-regulated channels is referred to as store-operated Ca^2+^ entry (SOCE). This influx of extracellular Ca^2+^ modulates numerous cellular events, such as cortical cytoskeleton reorganization, cell migration, the cell cycle, and the regulation of gene expression [reviewed in (9)]. This extensive regulation is attributed to the numerous Ca^2+^-dependent signaling pathways that rely on STIM1/ORAI1.

While the human *STIM1* gene (ENSG00000167323) has multiple transcriptional variants, its gene products have the same function as the canonical STIM1 protein (RefSeq NP_003147), i.e. the Ca^2+^ sensing and the regulation of Ca^2+^ channels (10). An additional function has been described for STIM1 in repressing the innate immune response. STIM1 has the ability to tether STING, a major inducer of the interferon response, to the ER, thereby preventing STING translocation to the ER-Golgi intermediate compartment and its activation (11). This novel function is mediated by the N-terminal domain of STIM1 and is linked to the activity of STIM1 as a Ca^2+^ sensor because the conformational change in STIM1 may underlie the reduced effectiveness of STIM1 as an ‘ER retention factor’ upon STIM1 activation due to Ca^2+^ store depletion (11,12).

STIM1 has a cytoplasmic domain that is still poorly studied, with large regions lacking defined functions. To fill this lack of knowledge we have carried out an analysis of the STIM1 interactome and have found numerous proteins related to nucleocytoplasmic trafficking and DNA repair. In this work, we have validated some of these interactions and identified a nuclear localization sequence (NLS) in STIM1 that mediates its transport to the inner nuclear membrane (INM). One of the STIM1 interactors that we have found is the Fanconi anemia (FA) protein FANCD2. FA, often referred to as FA/BRCA, is a genome instability disorder associated with developmental defects, bone marrow failure, and a predisposition to acute myeloid leukemias (AML), head-and-neck or breast cancers (13–16).

To date, 22 mutated genes have been identified in FA patients, and their gene products are named from FANCA to FANCW (17). One of the main functions of the FA/BRCA pathway is to repair DNA interstrand crosslinks (ICLs), which are covalent links between the two strands of the DNA that can distort the DNA helix and block the separation of the two DNA strands. This mechanical constraint blocks replication and transcription, and results in a slowing down of the cell S-phase and the accumulation of cells in G2/M phase because of unreplicated DNA caused by unrepaired ICLs. According to the current model, which is based on *in vitro* data, ICL repair by the FA pathway only occurs after a collision between two replication forks (18). The first detectable event in this repair is the active unloading of the stalled CMG helicases, a step dependent on ubiquitin signalling and the BRCA1-BARD1 tumour suppressor complex (19). Then, ubiquitylated FANCD2/FANCI localizes to the ICL and promotes dual incisions on either side of the ICL (referred to as ‘unhooking’) by recruiting of the SLX4 nuclease complex (20). The ICL unhooking generates a two-ended double strand break (DSB) and a DNA adduct, both of which are repaired by the cooperation of homologous recombination, translesion DNA polymerases, and nucleotide excision repair (NER), reviewed in (21). The FA repair pathway is widely considered as the main ICL repair pathway, though this has been challenged by some data obtained from cells in which fork convergence at an ICL comprises less than 20% of the observed events, with traverse and single fork collisions representing the majority of the observed events (22–24).

To date, there is no reported relationship between STIM1 and DNA repair pathways. However, this study proposes a novel non-canonical function for STIM1: the regulation of DNA damage repair, including the damage caused by ICLs-inducing agents. This newly discovered function is supported by the description of a set of interactors, such as FANCD2, FANCI, importins, as well as by the spatial regulation of STIM1 in response to mitomycin-C (MMC), an ICL-inducing agent. In addition, STIM1 has been found to accumulate at the nuclear envelope as well as in the chromatin-associated fraction of proteins, in response to MMC. Moreover, STIM1-KO cells showed enhanced endogenous DNA damage and greater sensitivity to MMC, hydroxyurea (HU), and aphidicolin, when compared to wild-type cells.

## Materials and methods

### Reagents

#### Chemicals

Importazole (#401105), benzonase (#E1014), hydroxyurea (#H8627), and Duolink In Situ Detection Reagents (#DUO92008) were purchased from Merck Life Science (Madrid, Spain). Mitomycin-C (sc-3514B), aphidicolin (sc-2201535), and rapamycin (sc-3504) were purchased from Santa Cruz Biotechnology (Bergheimer, Germany). Dulbecco's modified Eagle's medium (DMEM) was purchased from ThermoFisher Scientific (Waltham, MA, USA). Collagen type I was purchased from Corning (Corning, NY, USA). Clarity Max™ Western ECL substrate was from Bio-Rad (Hercules, CA, USA).

#### Antibodies

The full list of antibodies used in this study is provided in the Supplementary Table S1.

Secondary HRP-labelled antibodies were from Pierce (ThermoFisher Scientific, Waltham, MA, USA). Chromotek GFP-Trap agarose beads were purchased from Proteintech (Planegg-Martinsried, Germany).

### Biological resources

#### Cell lines

Flp-In™ HEK293 T-REx cells were purchased from ThermoFisher Scientific. U2OS cells were a kind gift from Dr Gopal Sapkota (University of Dundee), and they were modified to be stably transfected with the Flp-In™ system. U2OS cells edited by CRISPR/Cas9 to knock-out STIM1 or ORAI1 expression (STIM1-KO or ORAI1-KO U2OS cells) were generated and characterized elsewhere (25). Mycoplasma contamination was monitored with the mycoplasma Gel Detection Kit (Ref. #4542 from BioTools (Madrid, Spain) and all cell stocks used in this work were tested and found negative for this contamination.

#### DNA constructs

The vector for the inducible expression of STIM1-GFP or Flag-STIM1 was generated by inserting the cDNA of the human STIM1 isoform 2 (RefSeq accession NM_003156.4) into the *Bam*HI–*Not*I sites of the pcDNA5/FRT/TO-GFP or the pcDNA5/FRT/TO-Flag vector, both described in (26). Subsequent site-directed mutagenesis was performed with the QuikChange Multi Site-Directed Mutagenesis Kit (Agilent Technologies, Santa Clara, CA, USA) or by overlap extension PCR for large insertions or deletions. The construct for the expression of mouse STIM1-GFP has been described and characterized elsewhere (26,27). The construct for the stable and inducible expression of GFP-RAN (#DU31985) is available from https://mrcppureagents.dundee.ac.uk/. Directed mutagenesis to express GFP-RAN Q69L was performed as described above.

For FKBP12-FRB dimerization assays, the coding sequence of the human FKBP rapamycin binding (FRB) domain of mTOR was cloned between STIM1 and GFP coding sequences, using the *Not*I site, in the pcDNA5/FRT/TO-GFP vector. In addition, two additional copies of the FRB domain of mTOR were cloned after the GFP coding sequence, using an *Xho*I site, to facilitate the rapamycin-induced dimerization. FK506 binding protein (FKBP12) coding sequence was cloned into a pCMV5 plasmid, adding a (3×)PAAKKKKLD sequence that acts as a NLS (28) at the N-terminus of FKBP12. The cDNA of mCherry was cloned at the C-terminus of FKBP12 using *Eco*RI and *Not*I sites.

All DNA constructs were verified by DNA sequencing at the Sequencing Facility of the Universidad de Extremadura. DNA constructs used for transfections were purified from *Escherichia coli* DH5α using ZR Plasmid kits (Zymo Research, Irvine, CA, USA). Transfection of cells with DNA constructs was performed with 1.5 μg plasmid DNA per 10-cm dish and polyethyleneimine (Polysciences Inc., Eppelheim, Germany) in serum-containing medium, except for the generation of stable cell lines with pcDNA/FRT/TO vectors, where 0.5 μg construct + 4.5 μg of the pOG44 Flp recombinase expression vector were used.

### Generation of stable cell lines and CRISPR/Cas9 genome editing

Flp-In T-REx HEK293 cells, able to inducibly express tagged STIM1, were generated as described elsewhere (26,27). Briefly, cells were transfected with a mixture containing the cDNA in the pcDNA5-FRT/TO vector and the pOG44 vector at a 1:9 ratio using polyethyleneimine. After 48 h, the medium was replaced with a medium supplemented with 100 μg/ml hygromycin B + 15 μg/ml blasticidin to select stably transfected cells. HEK293 cells were cultured on 10-cm diameter dishes using Dulbecco's modified Eagle's medium (DMEM) with 10% (v/v) fetal bovine serum, 2 mM l-glutamine, 100 U/ml penicillin, 0.1 mg/ml streptomycin, 100 μg/ml hygromycin B, and 15 μg/ml blasticidin. The cells were maintained in a humidified atmosphere of 95% air/5% CO_2_ at 37°C. Prior to assays, cells were treated with 1 μg/ml doxycycline for 22–24 h to induce expression of tagged STIM1.

Genome editing to knock-out the expression of the human STIM1 in Flp-In T-Rex HEK293 cells (gene RefSeq accession NG_016277.1) was performed following established procedures used for other cell lines (25,29,30). Briefly, exon 5 was selected as the CRISPR target site and the guide pair (sense 5′-(G)AGATGACAGACCGGAGTCAT and antisense 5′-(G)AGTCCCTGTCATGGTGGTGT) were identified using the Sanger Institute CRISPR webtool (https://wge.stemcell.sanger.ac.uk//find_crisprs). Complementary oligos with *Bbs*I compatible overhangs were designed according to the Zhang method (31). These dsDNA guide inserts were then ligated into *Bbs*I-digested target vectors. The antisense guide was cloned into the spCas9 D10A expressing vector pX335 (Addgene Plasmid #42335), while the sense guide into the puromycin selectable plasmid pBABED P U6 (University of Dundee). The resulting constructs have been used in previous studies to successfully knock-out the *STIM1* locus in U2OS cells (25), PC3 cells (29) and SH-SY5Y cells (30). These constructs (#DU52282 and #DU52301) are available from https://mrcppureagents.dundee.ac.uk/. HEK293 cells were co-transfected with 1 μg of each plasmid using polyethyleneimine in a 10-cm dish. After 24 h of recovery and an additional 48 h of puromycin selection (2 μg/ml), the cell pool was sorted by FACS, and clones were subsequently analyzed for STIM1 depletion by immunoblotting. Genomic DNA from selected clones was isolated, and the region surrounding the target site of the guide RNAs at exon 5 was amplified by PCR (forward primer: 5′-CAAGAGCTAGAAGTGTTCCTGGG; reverse primer: 5′-CTTTGGTTTCCATGGCACAGC). The resulting PCR products were subcloned using the StrataClone Blunt PCR Cloning Kit (Agilent Technologies) and 10 colonies were picked and sequenced to verify the presence of indels. Sequencing data revealed a 47 bp deletion, thus confirming the successful KO of the *STIM1* locus (Supplementary Figure S1).

### Mass spectrometry analysis of STIM1 interactome

#### Preparation of samples and proteolytic digestion ‘on beads’

STIM1-GFP was pulled-down from HEK293 cells stably expressing STIM1-GFP (mouse STIM1, RefSeq accession NP_033313.2), as described elsewhere (26,27,32,33). Cells were lysed using the following buffer: 50 mM Tris–HCl (pH 7.5), 1 mM EGTA, 1 mM EDTA, 1 mM DTT, 1% (w/v) Nonidet P40 (NP-40), 1 mM sodium orthovanadate, 50 mM NaF, 5 mM sodium pyrophosphate, 0.27 M sucrose, 0.1% (v/v) 2-mercaptoethanol, 1 mM benzamidine and 0.1 mM phenylmethylsulphonyl fluoride (PMSF) (whole cell lysate buffer). Samples were lysed with 0.5 ml of ice-cold lysis buffer/10-cm dish, sonicated with 4 × 10-sec pulses with a setting of 40% amplitude using a Branson digital sonifier, and then centrifuged at 20 000 × *g* for 20 min (4°C). Protein concentration was determined using the Coomassie Protein Assay Reagent (ThermoFisher Scientific). Equilibrated GFP-Trap agarose beads (8 μl) were added to 1 mg of the clarified cell lysate. Lysates were incubated with the beads for 1 h at 4°C with gentle shaking. The agarose beads were washed three times with 1 ml lysis buffer containing 0.15 M NaCl and twice with buffer A (50 mM Tris–HCl (pH 7.5) and 0.1 mM EGTA). Agarose beads were resuspended in 2 M urea + 100 mM ammonium bicarbonate buffer and stored at −20°C. On-bead digestion was performed from the supernatants. Four biological replicates were digested with Lys-C (Alpha Laboratories) and trypsin (Promega) on beads as previously described (34) and desalted using StageTip (35).

#### MS analysis

Peptides resulting from all digestions were separated by nanoscale C18 reverse-phase liquid chromatography using an EASY-nLC II 1200 (Thermo Scientific) coupled to an Orbitrap Q-Exactive HF mass spectrometer for the in-gel digestion, or to an Orbitrap Fusion Lumos for the on-beads digestion (Thermo Scientific). For both instruments a nanoelectrospray ion source (Sonation) was used for ionization in positive mode. Chromatography was carried out using a 50 cm (on-beads digestion) fused silica emitter (CoAnn Technologies) packed in house with reverse phase Reprosil Pur Basic 1.9 μm (Dr Maisch GmbH). For the full scan a resolution of 60 000 at 250 Th was used. The top ten most intense ions in the full MS were isolated for fragmentation with a target of 100 000 at a resolution of 15 000 at 250 Th. MS data were acquired using the XCalibur software (Thermo Fisher Scientific). An Active Background Ion Reduction Device (ABIRD, ESI solutions) was used to decrease air contaminants signal level.

#### Data analysis

The MS Raw data were processed with MaxQuant software (36) version 1.6.3.3 and searched with Andromeda search engine (37) querying SwissProt (38) *Homo sapiens* (42438 entries). First and main searches were performed with precursor mass tolerances of 20 ppm and 4.5 ppm, respectively, and MS/MS tolerance of 20 ppm. The minimum peptide length was set to six amino acids and specificity for trypsin cleavage was required. Cysteine carbamidomethylation was set as fixed modification, whereas methionine oxidation, phosphorylation on serine-threonine-tyrosine, and N-terminal acetylation were specified as variable modifications. The peptide, protein, and site false discovery rate (FDR) was set to 1%. All MaxQuant outputs were analyzed with Perseus software version 1.6.2.3 (39).

Protein abundance was measured using label-free quantification algorithm available in MaxQuant (40) and reported in the ProteinGroups.txt file. Reverse and contaminant hits were removed, and only protein groups identified with at least one unique peptide were allowed in all lists of identified proteins. Only proteins robustly quantified in all three replicates in at least one group, were allowed in the list of quantified proteins. Missing values were imputed separately for each column, and significantly enriched proteins were selected using a permutation-based unpaired t-test with FDR set at 1%, comparing all samples to GFP. The results are summarized in the Supplementary Table S2.

### Immunoblot

Clarification of lysates were performed as indicated above for the preparation of MS analysis. Lysates (10–40 μg) were subjected to electrophoresis on polyacrylamide gels and subsequent electroblotting to nitrocellulose membranes. Membranes were blocked for 1 h at room temperature (RT) in blocking buffer: TBS-T (Tris-buffered saline buffer, pH 7.5, with 0.2% Tween-20) containing 10% (w/v) non-fat milk. Then, the membranes were incubated overnight with the specific antibody (see Supplementary Table S1), washed with TBS-T, and then incubated with anti-IgG horseradish peroxidase (HRP)-conjugated secondary antibodies (1:10 000 dilution in all cases) for 1 h at RT. Clarity Max™ Western ECL substrate was added to the membranes and the signal recorded with ChemiDoc XRS+ system (BioRad, Hercules, CA, USA). The recorded signal was quantified by volumetric integration using the Image Lab software (BioRad).

For the detection of ubiquitylated FANCD2, whole cell lysates were prepared with RIPA buffer (150 mM NaCl, 1% Triton X-100, 0.5% sodium deoxycholate, and 0.1% SDS), supplemented with 0.1% (v/v) 2-mercaptoethanol, 1 mM benzamidine, 0.1 mM phenylmethylsulphonyl fluoride (PMSF) and 10 mM iodoacetamide. Other conditions were as described above for whole cell lysate preparation.

### Co-immunoprecipitation analysis

To study the interaction of proteins with STIM1-GFP, tagged STIM1 (human STIM1, NP_003147.2) was pulled-down with GFP-Trap beads, as described above for the preparation of STIM1 interactome by MS analysis. After the final washing step with buffer A, proteins were eluted from the GFP-Trap beads by the addition of 15 μl NuPAGE-LDS sample buffer + 2.5% β-mercaptoethanol to the beads. Eluted proteins were heated at 90°C for 4 min and analyzed by immunoblot, as indicated above.

To immunoprecipitate endogenous STIM1, 2.5 mg HEK293 cell lysate was clarified with 3 × 15-min incubations of the lysate with 10 μl protein A/G-agarose beads and gentle rotation at 4°C. Clarified lysates were incubated with 5 μl anti-STIM1 antibody (Cell Signaling Technology, #5668) in binding buffer (10 mM NaPO_4_ pH 7.0, 140 mM NaCl, 0.05% Triton X-100) overnight at 4°C with gentle rotation. Then, 22 μl Dynabeads Protein A (Invitrogen #10001D) were added to the lysates and incubated for 1 h at 4°C. Dynabeads were washed with binding buffer 3 times, precipitated with a DynaMag rack, and eluted in 20 μl NuPAGE-LDS sample buffer + 2.5% β-mercaptoethanol. Eluted proteins were analyzed by immunoblot. Negative controls in the absence of specific antibody were carried out with normal rabbit IgG (Cell Signaling Technology #2729).

### Preparation of whole nuclear lysates

The subcellular fractionation to isolate cytoplasmic and nuclear proteins was adapted from (41). Briefly, cells (5–10 × 10^6^) were washed with PBS and lysed in 0.5 ml of CSK buffer A (340 mM sucrose, 10 mM KCl, 1.5 mM MgCl_2_, 10 mM HEPES, pH 7.9, 0.1% Triton X-100, 10% glycerol, 1 mM PMSF, 1 mM DTT, 1 mM benzamidine) for 10 min on ice. Cell lysate was centrifuged at 1300 × *g* for 5 min at 4°C to separate cytoplasmic proteins (supernatant) and nuclei (pellet). Nuclei were lysed in CSK buffer B (3 mM EDTA, 0.2 mM EGTA, pH 8.0, 1 mM DTT, 1 mM PMSF, 1 mM benzamidine) and were incubated for 10 min on ice (at a ratio of 500 μl buffer:100 μl pellet volume). Finally, nuclei were sonicated and clarified as indicated above for whole cell lysates.

### Preparation of nuclear envelope

Nuclear envelopes were prepared by the method described by Dreger *et al.* (42). Briefly, the nuclei (5–8 mg of protein) were suspended in 25 ml of ice-cold TP buffer (10 mM Tris–HCl, pH 8.0, 10 mM Na_2_HPO_4_, 1 mM PMSF, 1 mM benzamidine) containing 250 μg/ml of heparin, 1 mM Na_3_VO_4_, 10 mM NaF and 400 units of benzonase (ThermoFisher Scientific). The suspension was stirred for 90 min at 4°C. Nuclear envelopes were then pelleted by centrifugation at 10 000 × *g* for 30 min at 4°C, and resuspended in STM 0.25 buffer (50 mM Tris–HCl, pH 7.5, 0.25 M sucrose, 5 mM MgCl_2_, 2 mM DTT, 1 mM PMSF, 1 mM benzamidine) at ∼0.2–0.5 mg/ml.

### Preparation of endoplasmic reticulum lysates

The process used to isolate proteins associated with the ER was adapted from a previously published protocol (43). In brief, cells were washed with ice-cold PBS and harvested in 2 ml of PBS. After a 5-min centrifugation at 500 × *g*, the cell pellets were resuspended in 2 ml of ice-cold PBS and centrifuged at 1800 × *g* at 4ºC for 10 min. Cells were then resuspended in 2 ml of MTE buffer (containing 10 mM Tris–HCl, pH 7.4, 270 mM d-mannitol, 0.1 mM EDTA, 0.1 mM PMSF, 1 mM benzamidine) and the cell suspension was sonicated with 3 × 10-s pulses at 40% amplitude. Then, the lysates were centrifuged for 10 min at 15 000 × *g* at 4ºC. The resulting supernatant was layered over a discontinuous sucrose gradient (2, 1.5, 1.3 M sucrose) in MTE buffer and centrifuged at 152 000 × *g* for 70 min. A small volume (0.4–0.6 ml) of the large white band at the 1.3 M sucrose layer was extracted and mixed with 7 volumes of MTE buffer to dilute sucrose. Finally, the samples were ultracentrifuged at 126 000 × *g* for 45 min, and the pellet containing the ER extracts was resuspended in ∼100 μl of PBS.

### Isolation of chromatin-associated protein fraction

The isolation of proteins bound to chromatin was carried out following a previously described protocol (44) with minor modifications. Cells were washed with PBS at RT, scrapped in 1 ml of ice-cold PBS, and then centrifuged at 300 × *g* for 5 min. The resulting cell pellets were lysed in 5 volumes of lysis buffer (50 mM HEPES–KOH, pH 7.5, 140 mM NaCl, 1 mM EDTA, 10% glycerol, 0.5% NP-40, 0.25% Triton X-100, 1 mM DTT, 0.1 mM PMSF, 1 mM benzamidine) by gentle pipetting. Lysates were then centrifuged at 1100 × *g* for 5 min, and the supernatant was collected as the non-nuclear (cytoplasmic) fraction. The pellets were resuspended in an equal volume of the same lysis buffer, followed by another centrifugation at 1100 × *g* for 5 min. The new pellets were resuspended in the lysis buffer and incubated on ice for 10 min. After an additional centrifugation, the pellets were resuspended in 2 volumes of ice-cold washing buffer (10 mM Tris–HCl, pH 8.0, 200 mM NaCl, 1 mM EDTA, 0.5 mM EGTA, 0.1 mM PMSF, 1 mM benzamidine), and centrifuged at 1100 × *g* for 5 min. This washing step was repeated twice before nuclei were incubated on ice for 10 min. After a final centrifugation at 1100 × *g* for 5 min, the nuclei were resuspended in resuspension buffer (500 mM Tris–HCl, pH 6.8, 500 mM NaCl, 0.1 mM PMSF, 1 mM benzamidine). The nuclei were then sonicated with 4 × 10-s pulses at 40% amplitude using a Branson digital sonifier, and the samples were centrifuged at 20 000 × *g* for 20 min. The resulting supernatants, representing the chromatin-bound protein fraction, were saved and the protein concentration was measured as indicated above. All centrifugations were carried out at 4°C.

As an alternative method to validate the results, the proteins associated to the chromatin were isolated with the Subcellular Protein Fractionation kit for cultured cells (#78840 from ThermoFisher Scientific).

### FKBP12-FRB dimerization assay

STIM1-KO HEK293 cells were transfected for the inducible and stable expression of STIM1-FRB-GFP-(2x)-FRB. Stable cell lines were transfected for the transient expression of 3xNLS-FKBP12-mCherry. At this point, the expression of STIM1-FRB-GFP-(2x)-FRB was induced by addition of 1 μM doxycycline. Twenty-four hours later, cells were treated with 500 nM rapamycin for 10 min, and then whole nuclei lysates were prepared as described above.

In parallel, sibling plates were fixed with 4% paraformaldehyde in PBS for 10 min at RT. Coverslips were mounted onto glass slides using Hydromount (National Diagnostics). GFP and mCherry imaging was performed on a Nikon Ti-E inverted epifluorescence microscope with a Plan Apochromat 100× (NA 1.45) oil immersion objective. Cells were subjected to z-scanning (0.2 μm-step sections), and image deconvolution was carried out with 20 iterations and the 3D Landweber deconvolution method with NIS-Elements AR software (Nikon, Tokyo, Japan).

### Immunolocalization experiments

#### γH2AX, 53BP1, pBRCA1, FANCI and FANCD2 foci analysis

Cells were grown on collagen-coated coverslips for a minimum of 24 h and then treated with 40 ng/ml MMC for 18 h, 2 mM hydroxyurea (HU) for 6 h, or 0.2 μM aphidicolin for 48 h. U2OS cells were then permeabilized with a 2-min incubation with 0.5% Triton X-100 in PBS and then fixed with 4% paraformaldehyde for 10 min at RT. For HEK293, a 30-s incubation with 0.1% Triton X-100 in PBS at 4°C preceded fixation with 4% paraformaldehyde (also for 10 min at RT). In all cases, cells were blocked with 3% fish skin gelatin in PBS + 0.2% Tween-20 for 30 min at RT. Fixed cells were incubated overnight at 4°C with 1:1000 dilution of the following antibodies: rabbit anti-γH2AX (Cell Signaling Technology #9718), rabbit anti-FANCD2 (AbCam #ab108928), rabbit anti-53BP1 (Novus Biologicals #NB100-304SS), rabbit anti-pBRCA1 (Cell Signaling Technology #9009), and mouse anti-FANCI (Santa Cruz Biotechnology sc-271316). Coverslips were then incubated with secondary antibodies labeled with Alexa Fluor 488 or 594 at 1:1000 dilution for 20 min at RT. Finally, cells were incubated with 0.5 μg/ml Hoechst 33258 (#B-1155, Sigma-Aldrich) for 5 min, and coverslips were mounted onto glass slides using Hydromount (National Diagnostics). Images of fixed cells were taken on a Nikon Ti-E inverted epifluorescence microscope with a Plan Apochromat 100× (NA 1.45) oil immersion objective.

For co-localization experiments involving 53BP1, pBRCA1 and FANCD2 with γH2AX, the primary antibody was mouse anti-γH2AX (Merck #05-636) at a 1:1000 dilution. Anti-mouse IgG labeled with Alexa Fluor 594 and anti-rabbit IgG labeled with Alexa Fluor 488 were used as secondary antibodies (diluted 1:1000, 20 min at RT in the dark). All other conditions were consistent with those described above.

#### 53BP1 foci analysis in cyclin A2-negative cells

U2OS cells were cultured for 24 h and then 0.2 μM aphidicolin was added for 48 h. Cells were fixed with paraformaldehyde, permeabilized with 0.2% Triton X-100 in PBS for 10 min and blocked with 3% fish skin gelatin in PBS + 0.2% Tween-20 for 30 min at RT. Cells were incubated with anti-53BP1 (Novus Biologicals, NB100-304, diluted 1:1000 in blocking buffer), and anti-cyclin A2 (Proteintech 66391-1-Ig, 1:500 dilution in blocking buffer). Anti-mouse or rabbit IgG labeled with Alexa Fluor 488 or 594 were used as a secondary antibody (diluted 1:1000, 20 min at RT in the dark). Coverslips were mounted onto glass slides with Hydromount (National Diagnostics). Images of fixed cells were taken on a Nikon Ti-E inverted epifluorescence microscope with a Plan Apochromat 100× (NA 1.45) oil immersion objective. The number of 53BP1 foci/nucleus was evaluated in cyclin A2-negative cells only.

### Proximity ligation assay (PLA)

Proximity ligation assay (PLA) was carried out by using the Duolink In Situ Detection Reagents (#DUO92008). HEK293 cells, grown on collagen-coated coverslips, were fixed in 100% methanol for 15 min at −20ºC and washed 3 times with PBS. Following this, a 2-h blocking step was performed at 37°C using a blocking solution (3% fish skin gelatin in PBS + 0.2% Tween-20). Subsequently, cells were incubated overnight in a humidified chamber at 4°C with specific pairs of antibodies. The antibodies used for PLA assays were anti-GFP (sheep polyclonal antibody generated at University of Dundee, MRC-PPU Reagents and Services, fourth bleed, #S268B) at 1:100 dilution, mouse anti-FANCD2 (sc-20022 from Santa Cruz Biotechnology, 1:100 dilution), rabbit anti-FANCD2 (ab108928 from AbCam, 1:100 dilution), rabbit anti-STIM1 (#5668 from Cell Signaling Technology, 1:75 dilution), mouse anti-IPO8 (1:75 dilution), mouse anti-NUP205 (1:100 dilution), and mouse anti-FANCI (1:100 dilution). Following antibody incubation, the coverslips were washed three times with PBS + 0.2% Tween-20. Each coverslip was then incubated with 0.625 μl of each PLA probe (PLUS and MINUS) in a final volume of 50 μl of blocking solution for 1 h at 37°C. For the PLA probes, we used PLA probe anti-rabbit PLUS (#DUO92002), PLA probe anti-mouse MINUS (#DUO92004), PLA probe anti-goat MINUS (#DUO92006). After 3 washing steps with PBS + 0.2% Tween-20, 1.25 μl ligase in 1× ligation buffer (diluted in Milli-Q water, final volume of 50 μl) was added to each coverslip and incubated for 30 min at 37ºC. The ligation mix was removed with 3 washing steps, and each coverslip was incubated with 0.625 μl polymerase in 1× amplification red solution (diluted in Milli-Q water, final volume 50 μl) for 100 min at 37ºC. All subsequent steps were performed in the dark to avoid losing the fluorescent signal. To remove the amplification solution, coverslips were washed twice with 1× SSC buffer (#S6639 from Sigma-Aldrich) for 10 min each and once with 0.01 × SSC buffer for 1 min. Finally, cells were incubated with 0.5 μg/ml Hoechst 33258 (#B-1155, Sigma-Aldrich) for 5 min, and coverslips were mounted onto glass slides using Hydromount. Imaging was performed on a Nikon Ti-E inverted epifluorescence microscope with a Plan Apochromat 100× (NA 1.45) oil immersion objective.

### Super-resolution microscopy

Super-resolution images were acquired with Elyra 7 system (lattice SIM2) from Zeiss, equipped with two cameras sCMOS. The objective used was 63× (NA 1.4) oil immersion and Z step was set between 0.094 and 0.110 μm. Image processing was based in SIM algorithm from ZEN 3.0 SR FP2 software from Zeiss. 3D processing was made using Arivis Vision4D also from Zeiss.

### Colony formation assay

Two hundred cells were plated onto 60 mm-diameter dishes, and 24 h after plating, 5–20 ng/ml of MMC was added to the culture medium. Cells were cultured for 7–8 days, and the formation of colonies was visualized by fixing the cells with 4% paraformaldehyde (10 min at RT) and subsequently staining them with 0.1% crystal violet in 10% ethanol + PBS for 1 h, at RT. Excess of dye was washed out with water, and the plates were air-dried. The quantification of colony formation was performed by imaging the plates and analyzing the number of colonies with > 50 cells per colony. Colony count and size analysis were performed with the NIS-Elements AR software.

### Cell cycle distribution

Cells were plated and 7 h later 40 ng/ml MMC was added to the culture medium for an additional 18 h. The culture medium was then replaced with fresh medium, and the culture was extended for 48 h in the case of U2OS cells or 24 h for HEK293 cells. The cells were then trypsinized, centrifuged at 270 × *g* for 5 min, and the pellet was resuspended in PBS. After an additional washing step, cells were fixed by addition of ice-cold 70% ethanol for 5 min. Following a washing step in PBS + 1% BSA, cells were resuspended in PBS + 1% BSA with 0.5% propidium iodide and 50 μg/ml RNAse and incubated in the dark for 20 min with gentle agitation. Cell cycle analysis was performed by flow cytometry, with doublets discrimination and 20 000 cells per sample were acquired using a MACSQuant VYB flow cytometer (Miltenyi Biotech) at the Cytometry Facility of the Universidad de Extremadura. Flowlogic software (Inivai Technologies, Mentone, Australia) was used for quantitation of cell cycle phases.

### Comet assay

The analysis of DNA breaks was also monitored with the comet assay, as described in (45) with modifications. Cells were cultured in 10-cm dishes, and after 24 h, they were treated with 40 ng/ml MMC for 18 h. Thereafter, cells were harvested by trypsinization, and 50 000 cells were transferred to wells of a 24-well plate in 0.5 ml culture medium. Immediately, 1 ml of 1% low melting point agarose was mixed with the culture medium containing cells, and 1 ml of this mixture was placed onto a microscope slide pre-coated with 1% low EEO agarose. A rectangular coverslip was placed on top, and after 3–4 min, the coverslip was removed, and cells were lysed by dipping the slides in ice-cold lysis buffer (30 mM NaOH, 1 M NaCl, 0.1% N-lauryl sarcosine), followed by 2 washes with alkali buffer (30 mM NaOH, 2 mM EDTA) in the dark. Cells were subjected to electrophoresis for 15 min at 1V/cm/min in alkali buffer as a running electrophoresis buffer. Slides were washed once with neutralization buffer (1 M Tris–HCl, pH 7.5), and then washed twice with water. Cells were stained with 2.5 μg/ml propidium iodide for 5 min in the dark, followed with 3× 10-min washes in water with gently shaking. After air drying, slides were observed under the microscope using an S Fluor 20× objective (NA 0.75). Tail moment was quantified with the following calculation: DS3 × (((DS1 – DS2)/2) + (DS2/2)), where DS1 is the total comet length, DS2 is the comet head length, and DS3 is the fraction of DNA in the tail (measured using fluorescence intensities in the tail and head).

### Cytosolic free calcium concentration

Cytosolic free calcium concentration ([Ca^2+^]_i_) was measured in fura-2-AM-loaded cells as described elsewhere (25,46). Briefly, 170000 cells were plated on collagen-coated round coverslips and cultured for 36–48 h. Thereafter, cells were loaded with 1 μM fura-2-AM for 45 min in culture medium, washed with Hank's balanced salt solution (HBSS, #14025 from ThermoFisher Scientific), and placed on the DH-40i micro-incubation platform (Warner Instruments) of an inverted microscope Nikon Ti-E. All measurements were performed at 36°C. Excitation fluorescence wavelengths were selected with 340/26 and 387/11 nm filters (Semrock, Rochester, NY, USA), and emission fluorescence with a 510/10 nm filter. Cells were treated with 1 μM thapsigargin in Ca^2+^-free HBSS with the following composition: 138 mM NaCl, 5.3 mM KCl, 0.34 mM Na_2_HPO_4_, 0.44 mM KH_2_PO_4_, 4.17 mM NaHCO_3_, 4 mM Mg^2+^ and EGTA 0.1 mM (pH 7.4). After triggering Ca^2+^ store depletion with thapsigargin, store-operated Ca^2+^ entry (SOCE) was monitored with the addition of 2 mM CaCl_2_ to the assay medium, as described elsewhere (26,27). Data acquisition and analysis were performed with the NIS-Elements AR software.

### Statistical analysis of data

Statistical analyses between pairs of data groups were done using parametric or non-parametric unpaired *t*-test. Parametric *t*-tests were used when mean was calculated (immunoblots), whereas non-parametric unpaired *t*-tests (Mann–Whitney) were carried out when the distribution of data and the median were assessed (number of foci and tail moment). In all bar graphics, mean ± SEM is represented, in addition to the scattered data. Analyses were performed with the GraphPad software. Differences between groups of data were taken statistically significant for *P* < 0.05. The *P*-values are represented as follows: (*) *P* < 0.05, (**) *P* < 0.01, (***) *P* < 0.001, (****) *P* < 0.0001.

## Results

### STIM1 interacts with importins and is constitutively transported to the nucleus

**Figure 1.**
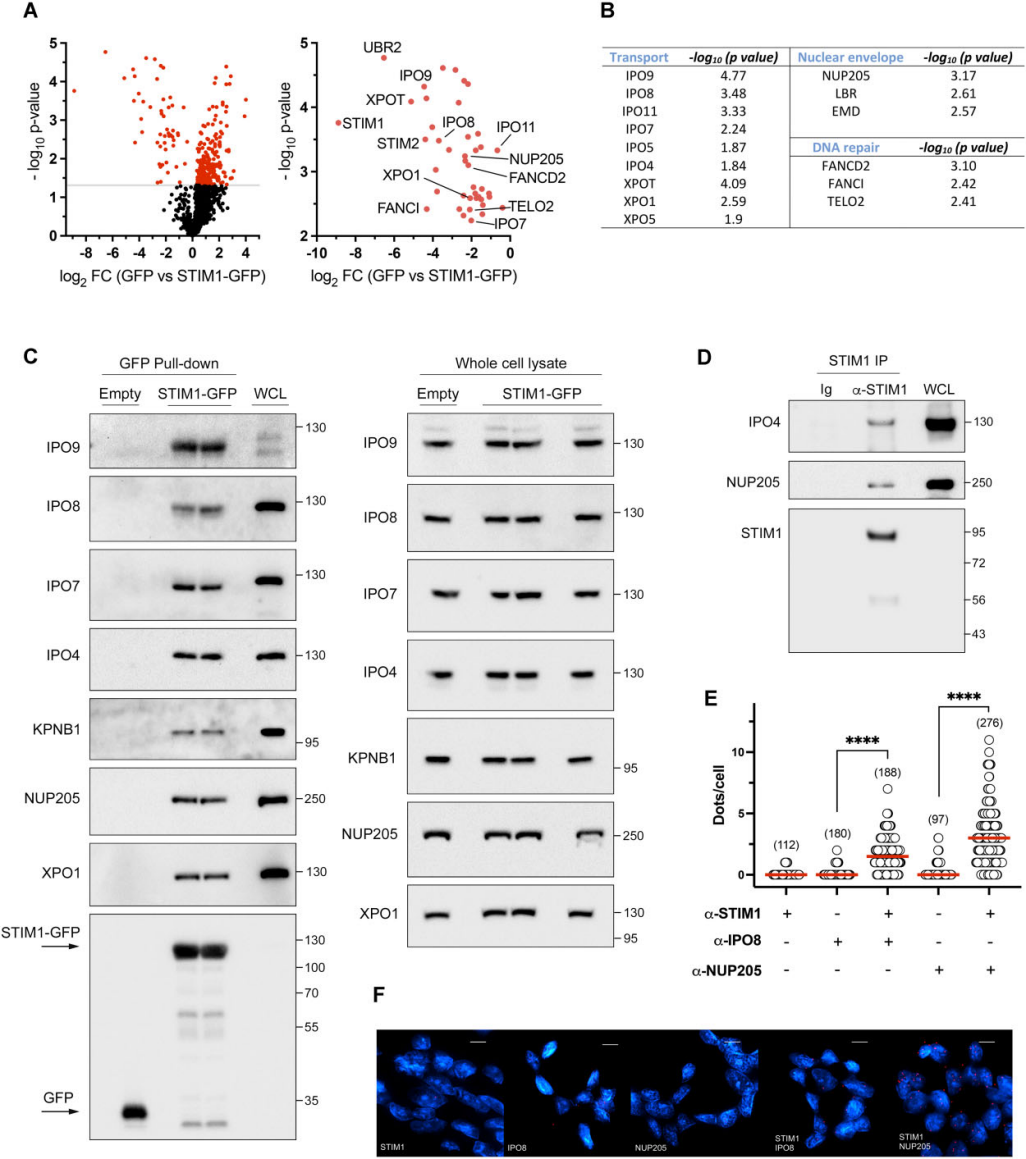
Identification of new STIM1 interactors. STIM1-KO HEK293 cells were stably transfected for the inducible overexpression of STIM1-GFP, or GFP as a negative control. GFP-tagged protein was pulled-down, the pellet was digested, and peptides were analyzed as described in the Methods section (*n* = 3 biological replicates). The results are shown in Supplementary Table S2. (**A**) *Left*: Volcano plot of the MS data. A significance threshold of *P* < 0.05 on y-axis was set to highlight potential interactors (–log_10_ Student's *t*-test *P*-value for GFP and STIM1-GFP values >1.3, shown as red symbols). *Right:* Re-scaled volcano plot to show interactors that bind STIM1-GFP with statistical significance when compared with GFP. (**B**) Statistical significance in the data from Supplementary Table S2 is summarized for some interactors with functions related to nucleocytoplasmic transport, interactors from the nuclear envelope, and DNA damage repair proteins. (**C**) *Left:* Co-immunoprecipitation assays were performed to validate interactions. STIM1-GFP or GFP expression was induced with 1 μM doxycycline for 22 h. Pull-down of GFP-tagged proteins was performed from whole cell lysates (WCL, 1 mg total protein in all cases, except for XPO1, where 0.25 mg of nuclear lysate was used), and co-precipitated proteins were analyzed by immunoblot. A fraction of WCL (3–5 μg) was loaded as a positive control. In all cases, blots are representative of three biological replicates. Levels of co-precipitated GFP were analyzed as a loading control, albeit only one blot for GFP is shown. *Right:* Total levels of potential interactors from whole cell lysates were evaluated by immunoblotting (30 μg protein/lane). (**D**) Endogenous STIM1 immunoprecipitation was performed from HEK293 WCL (2.5 mg protein), and a fraction of WCL (2.5 μg) was loaded as a positive control. Co-precipitated endogenous IPO4 and NUP205 were assessed as indicated above. Blots are representative of three biological replicates. (**E**) Interaction between STIM1 and IPO8 or NUP205 was assessed by proximity ligation assay (PLA) in HEK293 cells. Methanol-fixed cells were incubated with the specified antibodies (rabbit polyclonal anti-STIM1 antibody and mouse anti-IPO8 or mouse anti-NUP205). Negative controls were performed with single antibodies in independent assays. The number of red dots was assessed under fluorescence microscopy, with the number of cells evaluated indicated in parentheses. (**F**) Representative images for all conditions described in panel E. Scale bar = 10 μm.

To uncover possible undescribed functions of STIM1, we conducted a study of the STIM1 interactome using HEK293 cells expressing STIM1-GFP. We used genomic editing with CRISPR-Cas9(D10A) to generate a Flp-In T-REx STIM1-KO HEK293 cell line (characterized in Supplementary Figure S1a–c). We then transfected cells to achieve stable and inducible expression of STIM1 (full-length)-GFP (Supplementary Figure S1d) or -GFP only, as a negative control. After a GFP pull-down, the proteomic analysis of the hits revealed the presence of proteins that could potentially be novel STIM1 interactors (Supplementary Table S2). Among the potential interactors, a detailed analysis of the results indicated that STIM1 significantly interacted with numerous importins, including IPO4, IPO7, IPO8, IPO9, IPO11, as well as nuclear export proteins such as XPO1, XPO5 or XPOT. Additionally, STIM1 showed interactions with the nuclear pore complex protein NUP205 and the inner nuclear membrane protein emerin (Figure 1A, B and Supplementary Table S2).

To validate these results, we analyzed the co-precipitation of some of these proteins with STIM1 by immunoblotting. Using STIM1-deficient HEK293 cells (STIM1-KO) stably transfected for the inducible expression of STIM1-GFP, or GFP only as a negative control, we observed that STIM1 specifically co-precipitated with IPO9, IPO8, IPO7, IPO4, XPO1 and NUP205 (Figure 1C) in resting cells. We also assessed KPNB1, and while we found specific co-precipitation with STIM1-GFP, in this latter case, the co-precipitation was weaker than what was observed for other importins. Furthermore, interactions between STIM1 and IPO4 and NUP205 were corroborated through immunoprecipitation of endogenous STIM1 (Figure 1D), whereas the interaction of STIM1 with IPO8 and NUP205 was confirmed using a proximity ligation assay (PLA) (Figure 1E, F). These results suggest the possibility that STIM1 is transported to the nucleus via an importin-dependent mechanism.

**Figure 2.**
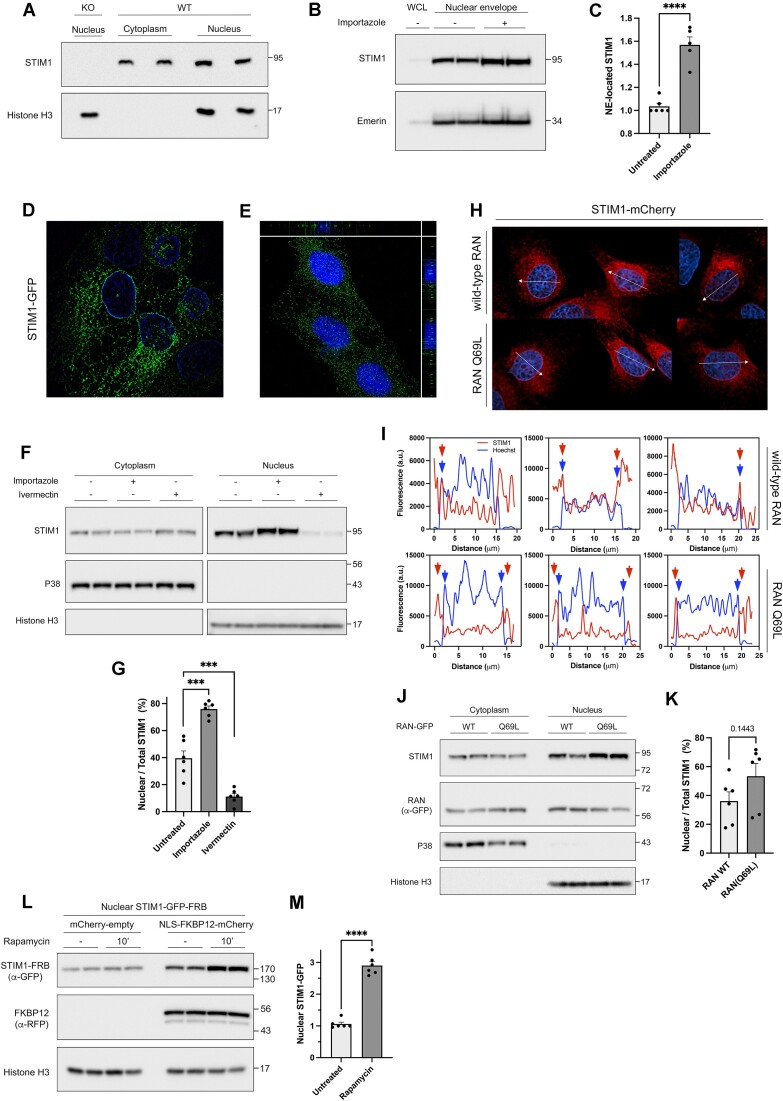
Assessment of nuclear levels of STIM1. (**A**) Total levels of endogenous STIM1 were analyzed in nuclear fractions (20 μg protein/lane) and non-nuclear fractions (10 μg protein/lane, labeled as *cytoplasm* in the figure) by immunoblotting. Histone H3 was used as a nuclear marker for control. (**B**) HEK293 cells were treated with 100 μM importazole for 4 h, and nuclear envelopes were then purified. Endogenous STIM1 levels were analyzed by immunoblotting (6 μg protein/lane), and emerin was assessed as a loading control. (**C**) Data from blots in panel B were quantified (*n* = 5 from two biological replicates) by calculating the volume of the STIM1 band relative to the volume of the emerin band. (**D**) An equatorial section of 0.094 μm z-section was acquired from U2OS cell stably expressing STIM1-GFP. A stack of sections with 3.4 μm in z-axis is shown in the Supplementary Video 1. (**E**) U2OS cells were fixed and endogenous STIM1 was immunolocalized using a rabbit anti-STIM1 antibody and a secondary antibody labeled with AlexaFluor 488. A stack of 0.094 μm z-sections is shown together with the orthogonal views of z-axis (x–z and y–z). (**F**) HEK293 cells were treated with 100 μM importazole for 4 h or with 50 μM ivermectin for 2.5 h. Cytosolic and nuclear fractions were assessed for endogenous STIM1 by immunoblotting (20 μg protein/lane in the cytosolic fraction and 40 μg for nuclei). P38 MAPK and histone H3 were analyzed as cytosolic and nuclear markers, respectively, and loading controls. (**G**) Quantification of data from panel F (*n* = 6, from two biological replicates) was carried out by calculating the volume of the cytosolic and nuclear STIM1 bands, and the ratio nuclear/total STIM1 as a percentage. (**H**) U2OS cells constitutively expressing STIM1-mCherry were transfected for the transient expression of GFP-RAN or GFP-RAN^Q69L^. Fixed cells were counterstained with Hoechst 33258 and observed under wide-field fluorescence microscopy. Only red and blue channels are shown. The distribution of RAN-GFP protein is shown in the Supplementary Figure S2c. (**I**) The intensity of fluorescence along the arrow in panel H (intensity profile) is shown for STIM1-mCherry and chromatin in an equatorial section of the nuclei. (**J**) Levels of RAN and endogenous STIM1 were assessed by immunoblotting in cytosolic and nuclear fractions from HEK293 cells stably expressing GFP-RAN or GFP-RAN^Q69L^ (20 μg protein/lane in cytosolic fraction and 40 μg for nuclei). (**K**) Quantification of data from panel J was calculated as described for panel G (*n* = 6, from three biological replicates). (**L**) HEK293 cells stably and inducibly expressing STIM1-FRB-GFP-^2x^FRB were transfected for the transient expression of ^3x^NLS-FKBP12-Cherry, or mCherry as a negative control. After 24 h, cells were treated with 500 nM rapamycin for 10 min. The localization of mCherry- and GFP-tagged proteins was assessed by epifluorescence microscopy (see Supplementary Figure S2e).Thereafter, nuclear fractions were isolated and assessed for the level of GFP-tagged STIM1, and the level of mCherry to assess transient transfection. Histone H3 was used as a loading control. (**M**) Quantification of data from panel L (*n* = 6 from three biological replicates) was performed by calculating the volume of the nuclear STIM1 band and expressing the result as relative to the amount of STIM1 in the absence of rapamycin.

STIM1 is an ER-resident protein, so it would require the interaction with importins for transport to the inner nuclear membrane (INM), similar to other proteins larger than approximately 40 kDa. To test this hypothesis, we performed fractionation of HEK293 cells to isolate nuclei and non-nuclear (cytoplasmic hereafter) fractions (Supplementary Figure S2a). This fractionation revealed the presence of a pool of endogenous nuclear STIM1 in resting cells (Figure 2A). To assess the active transport of STIM1 between the cytoplasm and nucleus, cells were treated with importazole, an inhibitor of importin recycling that disrupts the interaction between importin and the small nuclear GTPase RAN (47). Because the binding of RAN·GTP to importin is essential for disassembling the importin/cargo complex, the treatment with importazole leads to the accumulation of cargos within the nucleus. Importazole caused a significant increase in STIM1 levels at the nuclear envelope (Figure 2B, C), indicating active and constitutive transport of STIM1 into the nucleus in the absence of additional external stimuli. The quality of the nuclear envelope isolation was assessed by immunoblotting using markers of nuclear envelope, such as lamin B2, emerin, and NUP205, as well as markers of the chromatin fraction (histone H3), which is absent in the nuclear envelope fraction. Additionally, SERCA2 and VAPB served as markers for the ER (Supplementary Figure S2b), and P38 MAPK as a cytosolic marker.

It is crucial to emphasize that the accumulation of STIM1 in the nucleus is closely linked to its nature as a membrane protein and, therefore, it is not freely present in the nucleoplasm. However, super-resolution microscopy images of U2OS cells expressing STIM1-GFP (Figure 2D and supplementary Video 1), or immunolocalization of endogenous STIM1 (Figure 2E) demonstrate that a fraction of STIM1 is localized in the nuclear envelope, i.e. the fraction of endoplasmic reticulum (ER) that invaginates into the deeper regions of the nucleus, suggesting that in this manner, STIM1 may gain access to chromatin.

To further corroborate these findings, we treated cells with the importin pathway inhibitor, ivermectin, and observed a significant inhibition of STIM1 translocation to the nucleus (Figure 2F, G). This observation confirms the involvement of the classical importin pathway in this transport mechanism. Additionally, we expressed GFP-RAN and the dominant negative mutant RAN^Q69L^ to investigate the impact of RAN inhibition on STIM1 mobilization. Firstly, the microscopy images presented in Figure 2H reveal the presence of STIM1 within the nucleus, with a more pronounced presence at the periphery, where it overlaps with chromatin staining (see fluorescence profiles of three examples in Figure 2I) when wild-type RAN is expressed. The nuclear localization of GFP-RAN is further illustrated in the Supplementary Figure S2c, d. RAN^Q69L^, a mutant locked in the GTP-bound state, is known to associate with nuclear pore complexes (48). Consequently, it exhibits a distinct accumulation at the nuclear envelope, a localization which is also documented in this study (Supplementary Figure S2c, d). This accumulation of RAN at nuclear envelope leads to the build-up of RAN-dependent cargos, and we also observed an increase in STIM1 levels in nuclei when RAN^Q69L^ is expressed (Figure 2J, K). In addition, upon closer examination of the STIM1-mCherry fluorescence profiles (Figure 2H, I), it becomes evident that RAN^Q69L^ induces a separation between STIM1 and chromatin fluorescence signals, a result that supports that STIM1 accumulates at the outer nuclear envelope in these experimental conditions.

To further investigate the nuclear localization of STIM1, we used a strategy based on the rapamycin-induced dimerization of the 12-kDa FK506 binding protein (FKBP12) and the FKBP rapamycin binding (FRB) domain of mTOR. We induced the expression of the recombinant protein STIM1-FRB-GFP-2xFRB by adding doxycycline to a STIM1-KO HEK293 cell line that had been stably transfected for this purpose. Additionally, these cells were transfected for the transient expression of ^3x^NLS-FKBP12-mCherry, a variant of the protein with three copies of the SV40 nuclear localization signal (NLS) cloned at the N-terminus (see Methods), which localized in the nucleoplasm (Supplementary Figure S2e). STIM1-FRB-GFP-^2x^FRB showed the expected subcellular distribution throughout the ER (Supplementary Figure S2e). The treatment of cells with rapamycin (500 nM for 10 min) triggered the accumulation of STIM1-FRB-GFP-^2x^FRB in the nucleus of those cells that co-expressed ^3x^NLS-FKBP12-mCherry, but not in the absence of the bait (FKBP12) (Figure 2L, M). Together, all these findings demonstrate that STIM1 is transported to the nucleus, and that a fraction of STIM1 is localized at the inner nuclear membrane (INM).

### Identification of a nuclear localization signal (NLS) in STIM1

**Figure 3.**
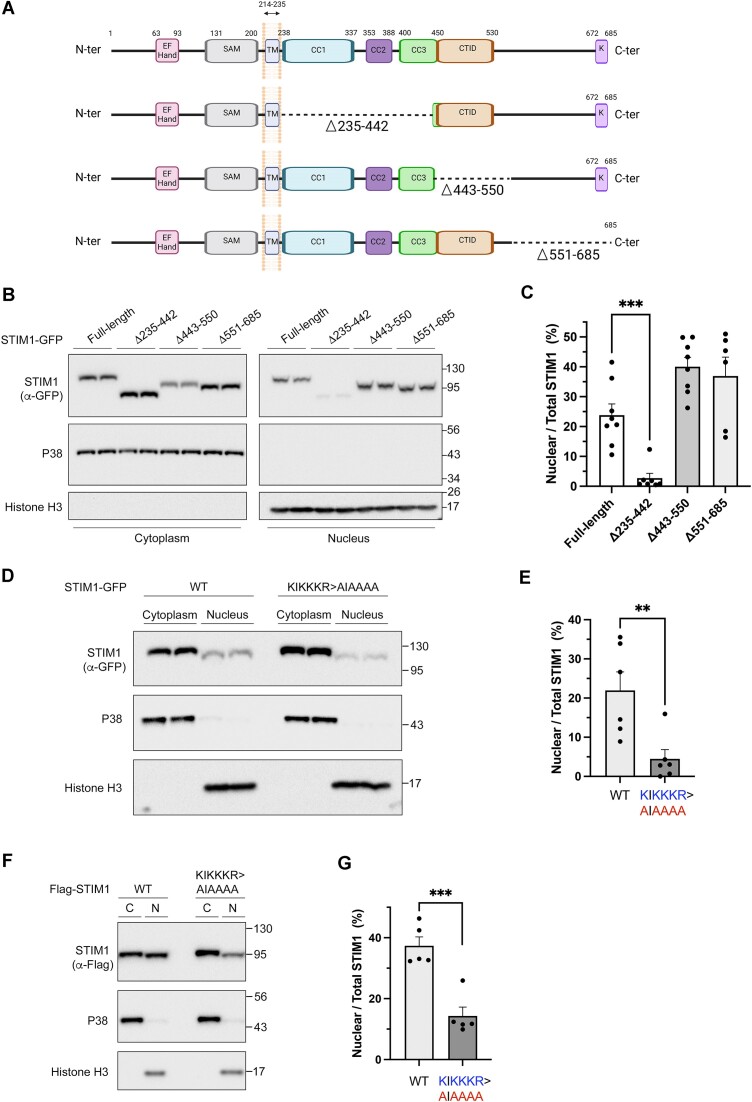
Identification of a nuclear localization signal in STIM1. (**A**) Functional domains identified in STIM1: Ca^2+^-sensitive EF-hand domain, sterile alpha motif (SAM), transmembrane domain (TM), coiled-coil domains (CC1-3), C-terminal inhibitory domain (CTID), and lysine-rich domain (K). Deleted sequences and domains in the C-terminal are indicated. This panel was created with BioRender.com. (**B**) STIM1-KO cells inducibly expressing truncated forms of STIM1 tagged with GFP, were used to prepare nuclear and non-nuclear fractions. Levels of STIM1 in both fractions were studied by immunoblotting (20 μg protein/lane in non-nuclear fractions, and 30 μg protein/lane in nuclear fractions). Levels of P38 MAPK (cytosolic marker) and histone H3 were assessed as loading controls and to evaluate the purification of fractions. Blots are representative of *n* = 2 biological samples (*n* > 6 total replicates). (**C**) The percentage of STIM1 in the nuclear fraction relative to the total STIM1 (nuclear + non-nuclear) is shown. (**D**) Levels of STIM1 were evaluated from non-nuclear (20 μg protein/lane) and nuclear fractions (30 μg protein/lane) isolated from cells expressing STIM1-GFP (labeled as WT) or a mutated version of the protein with the sequence ^382^AIAAAA^387^ replacing the wild-type ^382^KIKKKR^387^. P38 and histone H3 were assessed as a loading controls. (**E**) Quantification of data from blots shown in panel D was calculated as described for panel C (*n* = 3 biological replicates, and *n* = 6 total replicates). (**F**) STIM1 levels were assessed in non-nuclear (15 μg protein/lane) and nuclear fractions (30 μg protein/lane) from cells expressing Flag-STIM1 (WT) or Flag-STIM1(^382^KIKKKR^387^> ^382^AIAAAA^387^). P38 MAPK and histone H3 were used as cytosolic and nuclear markers, respectively. (**G**) Quantification of data from the blots in panel F was calculated as described for panels C and E (*n* = 2 biological replicates, and *n* = 5 total replicates).

Importins interact with cargos either through a NLS in the cargo sequence or via other mediators that harbor one or more NLSs. To determine whether STIM1 has a functional NLS, we studied the steady-state levels of STIM1 with deletions in its C-terminal domain (non-ER domain) (Figure 3A). The results revealed that the deletion of the fragment spanning amino acids 235–442 led to significant lower levels of nuclear STIM1 with the consequent accumulation of non-nuclear STIM1 in the cytosolic fraction (Figure 3B, C). Conversely, deleting sequences 443–550 or 551–685 increased steady-state nuclear STIM1 levels, a result that will be discussed in more detail later (see first paragraph of the Discussion section). Since NLSs typically consist of short sequences enriched in basic residues (49), we focused on the fragment 235–442, where we found that the sequence ^382^-KIKKKR-^387^ fits well to a canonical monopartite NLS. We next evaluated the role of this sequence as a NLS for STIM1 by generating the mutation ^382^-KIKKKR-^387^ to ^382^-AIAAAA-^387^. This modification significantly reduced STIM1 levels in the nucleus (Figure 3D, E), assessed by immunoblotting, suggesting that this sequence plays a critical role in the cytoplasm-to-nucleus transport of STIM1. To rule out any potential influence of GFP-mediated transport to the nucleus, we performed a parallel experiment, labeling proteins with Flag (Figure 3F, G). This experiment confirmed the requirement of the NLS described for the translocation of STIM1 to the nucleus.

**Figure 4.**
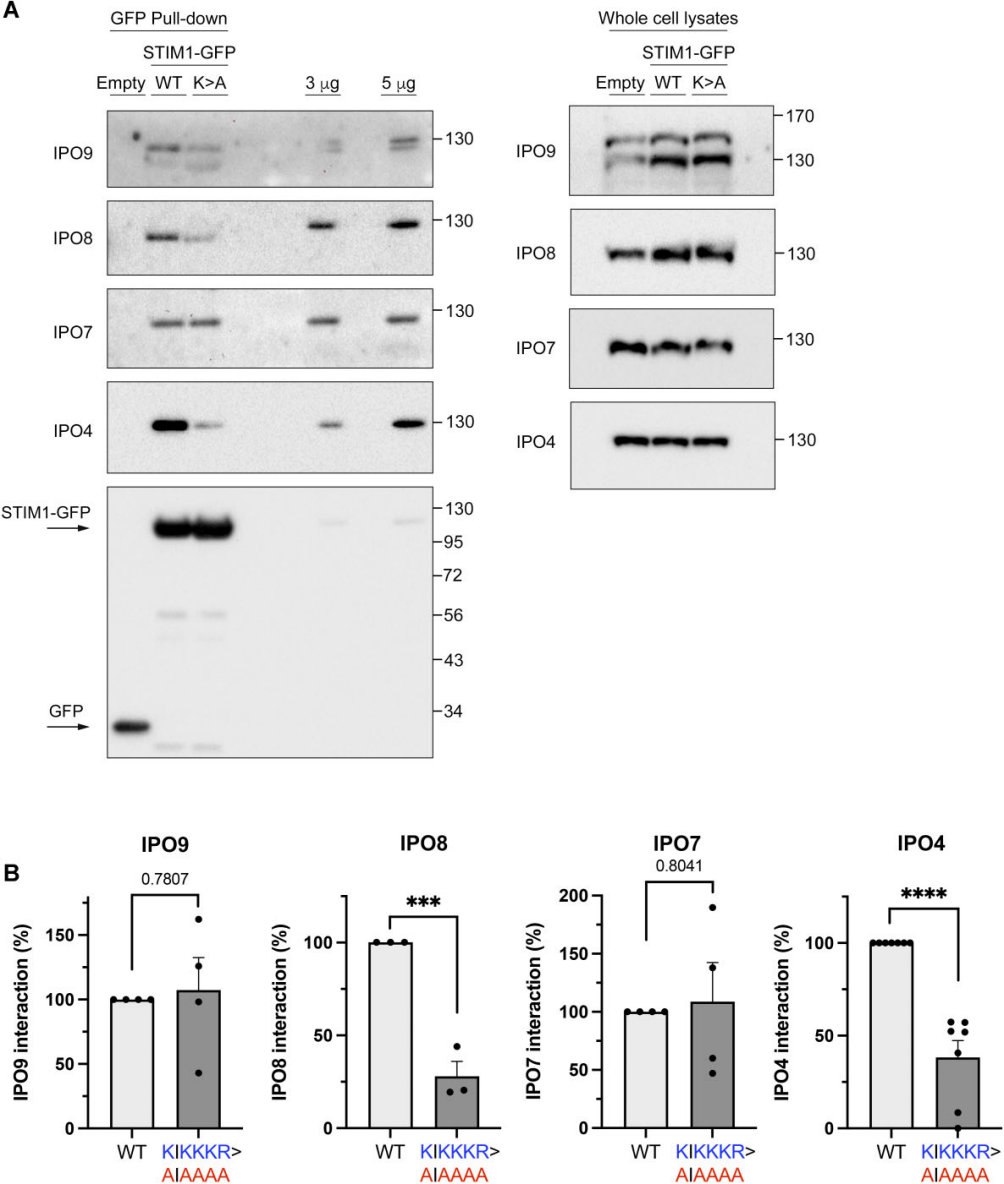
Mutation ^382^AIAAAA^387^ reduces co-precipitation with importins. (**A**) One milligram of whole cell lysates from STIM1-KO HEK293 cells inducibly expressing STIM1-GFP (labeled as WT), STIM1(^382^AIAAAA^387^)-GFP (labeled as K > A) or GFP only (labeled as empty) was used to pull-down GFP-tagged proteins and to assess co-precipitated IPO4, IPO7, IPO8 and IPO9 (left panels). Total pulled-down GFP was assessed as a control. A fraction of the lysates (30 μg protein) served as a loading control (inputs, right panels). (**B**) Quantification of data from panel A was performed by calculating the volume of the importin band relative to the volume of the STIM1-GFP band. Technical replicates: *n* = 7 for IPO4, *n* = 4 for IPO7, *n* = 3 for IPO8 and *n* = 4 for IPO9, from a minimum of three biological replicates in all cases.

In support of this, we also observed that the mutation of the sequence ^382^-KIKKKR-^387^ to ^382^-AIAAAA-^387^ (K > A in Figure 4A) significantly reduced the co-precipitation of STIM1 with importins IPO4 and IPO8, while the co-precipitation with IPO7 and IPO9 remained unaltered (Figure 4A, B). This result not only confirmed the role of this sequence as a *bona fide* NLS but also indicated that only IPO4 and IPO8 were dependent on this sequence for their interaction with STIM1. Because the mutation ^382^-KIKKKR-^387^ > ^382^-AIAAAA-^387^ reduced the levels of nuclear STIM1, these findings underscore to IPO4 and IPO8 as major regulators of this transport.

### STIM1 is a chromatin binding protein

**Figure 5.**
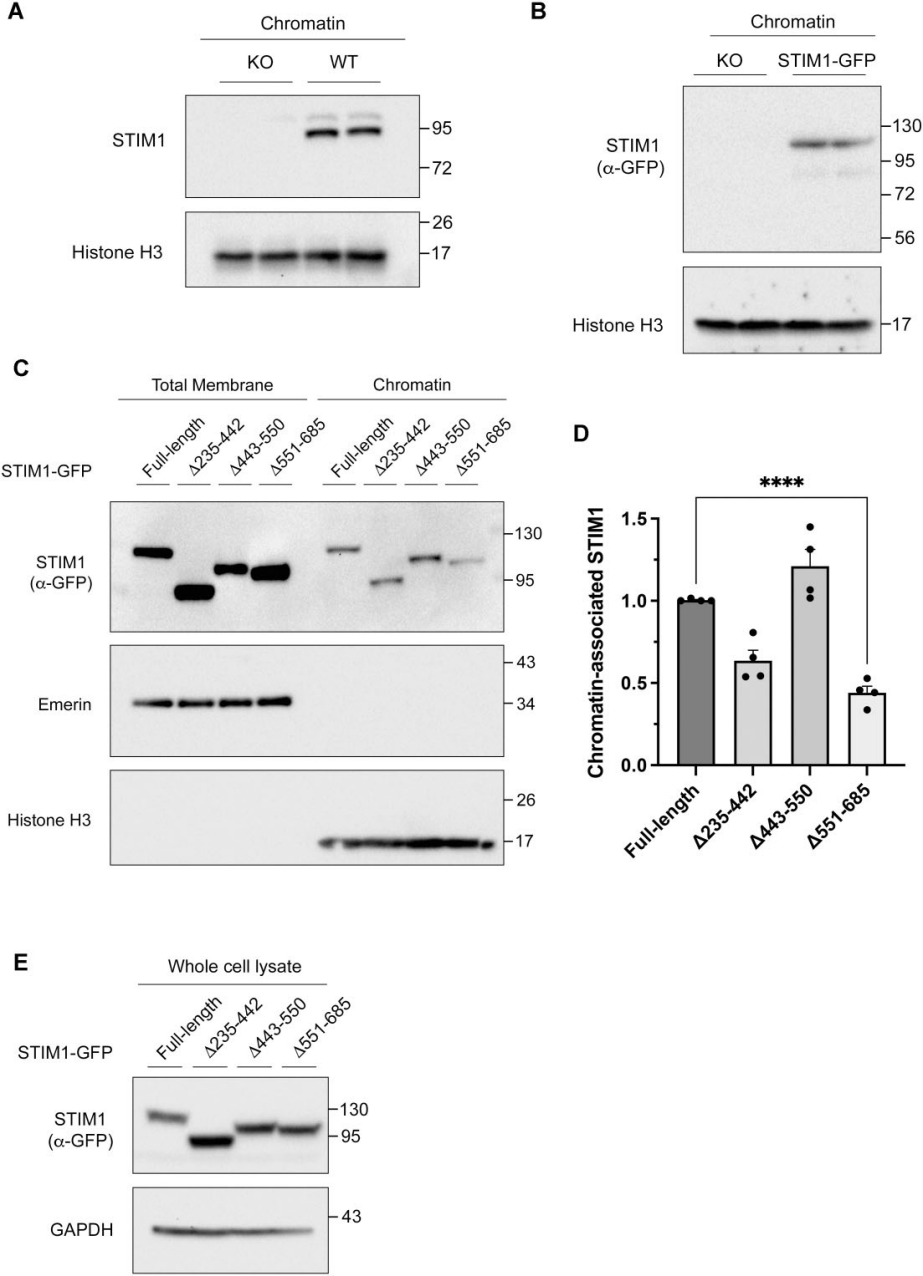
STIM1 is a chromatin-binding protein. Chromatin fractions were purified from HEK293 cells (panel **A**) or from STIM1-KO HEK293 cells inducibly expressing STIM1-GFP (panel **B**). After fractionation, 30 μg protein/lane were assessed by immunoblot. Histone H3 is shown as a loading control. Blots are representative of three biological samples and six total replicates. (**C**) STIM1-GFP constructs described in the Figure 3A were used to purify chromatin-bound proteins (right), or total membrane proteins (left), loading 40 and 20 μg protein/lane respectively. Full-length and truncated STIM1 were evaluated by immunoblotting. Emerin and histone H3 were used as membrane and chromatin markers, as well as loading controls. (**D**) Quantification of data from blots in panel C (*n* = 2 biological samples and *n* = 4 total replicates) was performed by calculating the chromatin-bound/total STIM1-GFP ratio, normalized to levels of total STIM1-GFP in whole cell lysates shown in panel E. (**E**) Total lysates (30 μg) were prepared from STIM1-KO HEK293 cells inducibly expressing full-length STIM1 tagged with GFP (STIM1-GFP), or versions of STIM1-GFP with the following deletions: 235–442, 443–550 or 551–685. Protein expression was analyzed by immunoblotting using an anti-GFP antibody (α-GFP) as primary antibody. As a loading control, levels of GAPDH were assessed.

The location of STIM1 at the INM, together with the observation that a subset of proteins closely related to DNA damage response (DDR) pathways, such as FANCD2 and FANCI, were identified as potential interactors of STIM1 (Supplementary Table S2 and Figure 1A), led us to hypothesize that STIM1 might function as a chromatin-associated protein with a role in DNA repair. Subcellular fractionation experiments confirmed that STIM1 is a chromatin-associated protein, a result observed for both the endogenous protein (Figure 5A) and overexpressed STIM1-GFP (Figure 5B).

Next, we searched for a DNA-interacting motif using the constructs described in Figure 3A. This strategy allowed us to determine that this domain is distal within the C-terminus of the protein (amino acids 551–685). The deletion of this segment resulted in a reduced level of STIM1 at the chromatin fraction (Figure 5C, D), while it had no impact on its nuclear transport (Figure 3B, C). This suggests that this specific fragment of STIM1 contains key residues or motifs that mediate the interaction with chromatin. In addition, we verified that the expression of this variant (Δ551–685) exhibited no alterations in total expression levels (Figure 5E), and its levels in the bulk ER and NE were similar to those observed for other variants (Supplementary Figure S3).

In addition, the deletion of the amino acids 235–442 yielded lower levels of STIM1 at the chromatin (Figure 5C, D). However, this result can be explained by a decreased amount of nuclear STIM1 resulting from the loss of the previously described NLS in the earlier experiments (see Figure 3).

### STIM1 interacts with proteins of the FA/BRCA pathway

**Figure 6.**
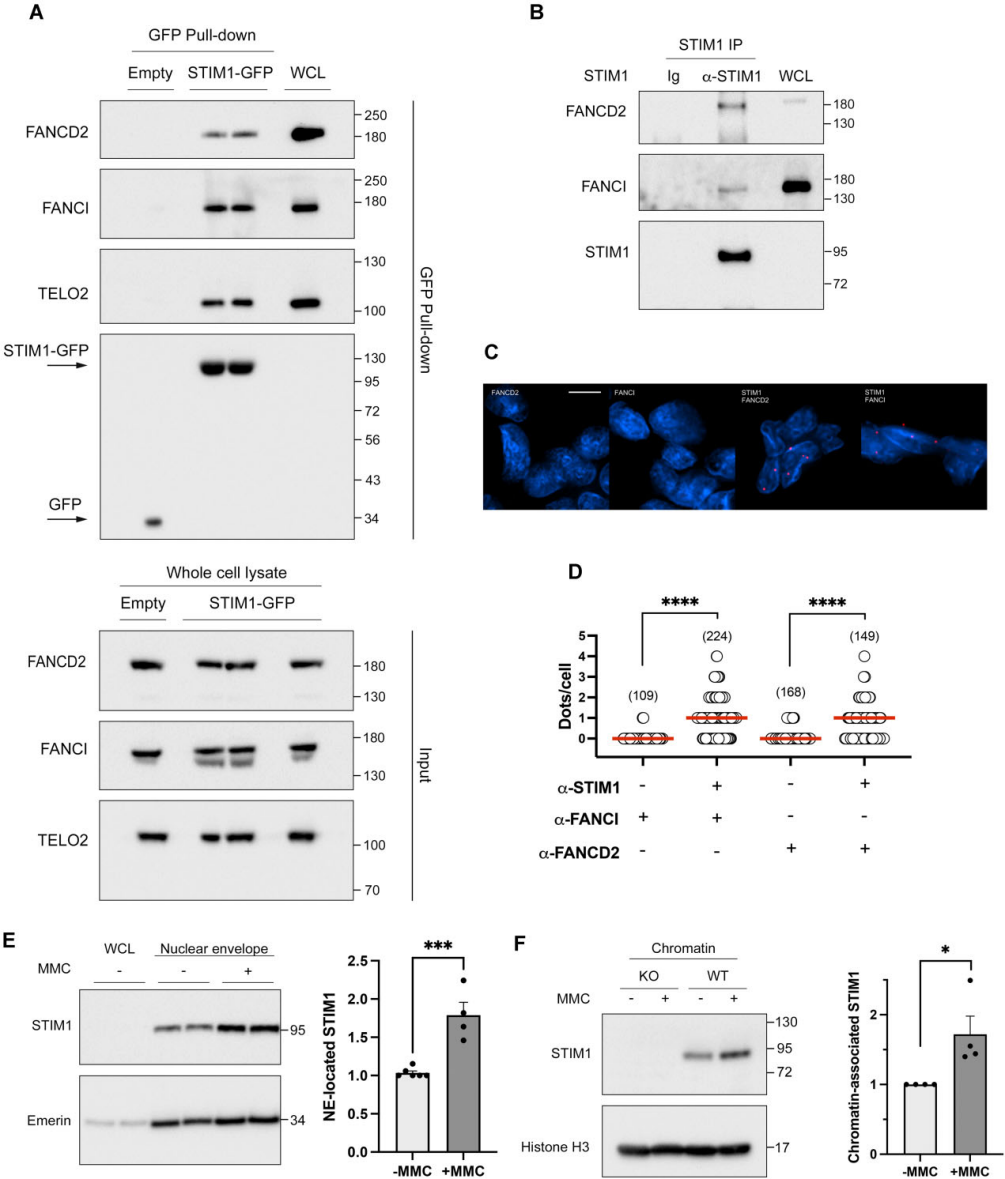
Validating interactions with DDR proteins. (**A**) *Top*: STIM1-GFP or GFP expression was induced with 1 μM doxycycline for 22 h. Pull-down of GFP-tagged proteins was performed from 1 mg of whole cell lysates (WCL), and co-precipitated proteins were analyzed by immunoblot. A fraction of WCL (3–5 μg) was loaded as a positive control. In all cases, blots are representative of three biological replicates. Co-precipitated GFP was assessed as a loading control. *Bottom:* Total levels of FANCD2, FANCI and TELO2 in WCL were evaluated by immunoblotting (input, 30 μg protein/lane). (**B**) Endogenous STIM1 immunoprecipitation was performed from WCL (2.5 mg total protein), and a fraction of this lysate (2.5 μg) was loaded as a positive control (labeled as WCL). Co-precipitated endogenous FANCD2 and FANCI were assessed as indicated above. Blots are representative of three biological replicates. (**C**) Interaction between endogenous STIM1 and FANCD2 or FANCI was assessed by PLA. Methanol-fixed HEK293 cells were incubated with the specified antibodies (rabbit polyclonal anti-STIM1 antibody and mouse anti-FANCD2 or mouse anti-FANCI). Red dots were examined under fluorescence microscopy, and representative images for all conditions are provided. (**D**) Quantification of data from panel C. The number of evaluated cells is indicated in parentheses. (**E**) *Left*: HEK293 cells were treated with 40 ng/ml MMC for 18 h, and then nuclear envelopes were isolated. Endogenous STIM1 was analyzed by immunoblotting (6 μg protein/lane). Emerin was assessed as nuclear envelope loading control. *Right*: Quantification of data from blots (*n* = 4 from two biological replicates). The quantification was carried out by calculating the volume of the STIM1 band relative to the volume of the emerin band, and normalizing values to untreated samples. (**F**) *Left*: HEK293 and STIM1-KO HEK293 cells were treated with 40 ng/ml MMC for 18 h, and then chromatin-associated proteins were extracted. Endogenous STIM1 level was analyzed by immunoblotting. Histone H3 was assessed as loading control. *Right*: Quantification of data from blots (*n* = 4 from three biological replicates).

The potential interaction of STIM1 with members of the FA/BRCA pathway, as suggested by our proteomics analysis (Supplementary Table S2), was assessed by classical co-immunoprecipitation with STIM1-GFP. The results confirmed a robust interaction between STIM1 and FANCD2, FANCI, and TELO2 (Figure 6A). The interaction between STIM1 and FANCD2 or FANCI at endogenous levels was observed through classical immunoprecipitation of endogenous STIM1 (Figure 6B) and by PLA (Figure 6C, D), thus confirming the findings from the STIM1 interactome analysis (Figure 1A, B and Supplementary Table S2).

Remarkably, STIM1 co-precipitated with both FANCD2 and FANCI, as both proteins form a heterodimer that binds to chromatin in response to DNA damage. Therefore, we hypothesized that STIM1 might exhibit a dynamic response to DNA damage induced with MMC, an agent known to induce the formation of ICLs in DNA, a modification that leads to the formation of DNA breaks (50). We observed that the treatment of cells with 40 ng/ml MMC for 18 h triggered an increase in STIM1 levels at the nuclear envelope (Figure 6E) and the chromatin fraction (Figure 6F), indicating that DNA damage is a stimulus that enhances the translocation of STIM1 to the nucleus. Based on these observations we hypothesized that STIM1 may play a role in the repair of DNA lesions within the FA/BRCA pathway.

### STIM1-deficient cells exhibit the three hallmarks of FA/BRCA-deficient cells

**Figure 7.**
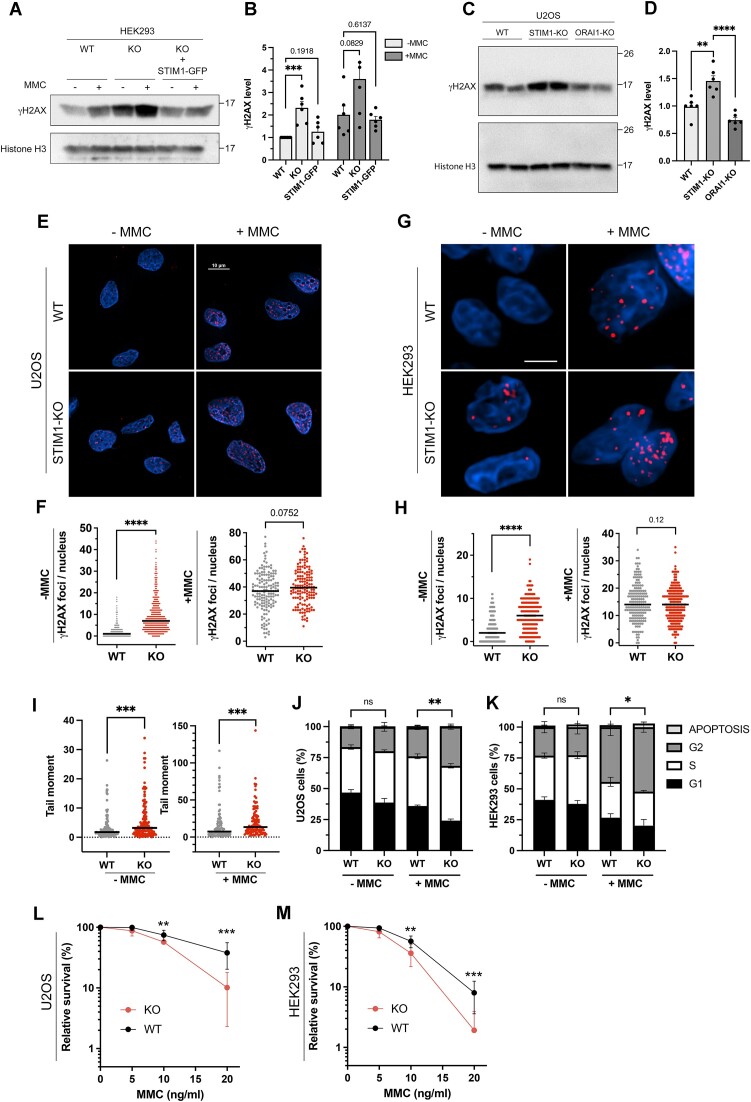
STIM1-deficient cells show enhanced basal DNA damage and higher sensitivity to mitomycin-C. (**A**) γH2AX levels in chromatin fractions from HEK293 cells, STIM1-KO cells, or STIM1-KO cells inducibly expressing STIM1-GFP for 22–23 h (20 μg protein/lane), treated with 40 ng/ml MMC for 18 h, or with the vehicle. (**B**) Quantification of data from blots shown in panel A was carried out by calculating the volume of the STIM1 band relative to the volume of the histone H3 band. Data are normalized to levels found in HEK293 cells non-treated with MMC (*n* = 3 biological samples and *n* = 6 technical replicates for WT and KO cells; *n* = 2 biological samples and *n* = 6 technical replicates for STIM1-GFP expressing cells). (**C**) γH2AX levels from nuclear fractions in the parental U2OS cell line (WT), STIM1-KO cells, and ORAI1-KO cells (20 μg protein/lane). (**D**) Quantification of data from blots shown in panel C. Data are normalized to levels found in U2OS WT cells (*n* = 3 biological samples and n = 6 total replicates). (**E**) Immunolocalization of γH2AX foci in permeabilized nuclei from U2OS and STIM1-KO U2OS cells treated with 40 ng/ml MMC for 18 h, or with the vehicle (bar = 10 μm). (**F**) Quantification of γH2AX foci in panel E (*n* = 2 biological samples and *n* = 4 total replicates. Cells analyzed: *n* = 437 (WT-MMC); *n* = 150 (WT + MMC); *n* = 483 (KO-MMC); *n* = 141 (KO + MMC). (**G**) Immunolocalization of γH2AX foci from HEK293 cells, treated as in panel E (bar = 5 μm). (**H**) Quantification of γH2AX foci in panel G (*n* = 2 biological replicates). Cells analyzed: *n* = 254 (WT-MMC); *n* = 172 (WT + MMC); *n* = 212 (KO-MMC); *n* = 223 (KO + MMC). (**I**) Alkaline comet assay was carried out using HEK293 and STIM1-KO HEK293 cells treated with 40 ng/ml MMC for 18 h, or with the vehicle (see Supplementary Figure S4 with representative images from this assay). Tail moment was quantified from *n* = 126 (WT); *n* = 112 (WT + MMC); *n* = 137 (KO); *n* = 107 (KO + MMC) (*n* = 2 independent experiments). (**J**, **K**) U2OS cells (panel J) or HEK293 cells (panel K) were treated with 40 ng/ml for 18 h and then cell culture was extended for 48 h (U2OS) or 24 h (HEK293) before cell sorting to analyze cell cycle distribution. Quantification of cell cycle distribution was performed from n = 6 biological replicates. (**L**, **M**) U2OS (panel L) and HEK293 cells (panel M) were plated and 24 h later 5–20 ng/ml MMC was added to the culture medium. After 7–8 days, colonies were stained with crystal violet. Colonies with >50 cells were considered for this calculation (*n* = 3 biological samples and *n* = 8 total replicates for U2OS, and *n* = 4 biological samples and *n* = 12 total replicates for HEK293 cells).

To test our hypothesis, we investigated whether STIM1-deficient cells displayed the three cellular hallmarks of FA/BRCA-deficient cells, i.e. DNA breaks, stress-induced G2/M cell cycle arrest, and sensitivity to ICLs. DNA breaks in STIM1-deficient cells were monitored by measuring the levels of γH2AX, a widely used marker of DNA breaks (51). Consistent with the potential role of STIM1 in the FA/BRCA pathway, the basal level of γH2AX was increased in STIM1-KO cells compared to the parental (wild-type) cell line. Additionally, a greater level of γH2AX was observed after a treatment with MMC (Figure 7A, B). The DNA damage observed in STIM1-KO cells was prevented by the ectopic overexpression of STIM1-GFP (Figure 7A, B), ruling out the possibility that the observed results were due to off-targets of the genome editing method. Importantly, the increased levels of γH2AX found in STIM1-KO cells could not be replicated in ORAI1-deficient cells (Figure 7C, D). This latter result excludes a role for Ca^2+^ entry through ORAI1 in response to DNA damage and suggests that STIM1 may have a non-canonical function that is independent of its role as an activator of the plasma membrane Ca^2+^ channel ORAI1.

To confirm the increased level of DNA breaks in STIM1-KO cells that we observed by immunoblotting, we monitored the formation of γH2AX foci by immunofluorescence in wild-type and STIM1-KO cells. We thus corroborated the existence of more extensive basal or endogenous DNA damage in STIM1-deficient cells (Figure 7E–H), whether U2OS or HEK293 cells. Furthermore, although the difference in lesion levels was statistically non-significant when exposed to MMC, there was a slight increase in STIM1-KO U2OS and HEK293 cells compared to the parental cell line. Although γH2AX is probably the main marker for DNA lesions, it is accepted that other mechanisms, such as apoptosis, can influence H2AX phosphorylation levels (52). Therefore, we also monitored DNA breaks using the comet assay, which confirmed the presence of an increased level of DNA breaks in STIM1-deficient cells, both with and without exposure to MMC (Figure 7I, and Supplementary Figure S4). These results are consistent with a role for STIM1 in the repair of DNA lesions.

We then monitored G2/M arrest following exposure to MMC. Treating U2OS cells and HEK293 cells with MMC to induce the formation of ICLs resulted in an increased number of cells at the G2/M phase of the cell cycle (Figure 7J, K). Notably, this arrest was more prominent in STIM1-deficient cells than in the parental cell line, for both cell types.

Finally, using the clonogenic survival assay to assess cell sensitivity to ICLs, we found that STIM1-deficient cells were significantly more sensitive to ICLs induced by MMC (Figure 7L, M) in both cell types, U2OS and HEK293 cells. In summary, our findings demonstrate that STIM1-KO cells exhibit three hallmarks of FA/BRCA-deficient cells, indicating that *STIM1* may be a novel candidate gene in the FA/BRCA pathway.

### Impaired DDR in STIM1-deficient cells

**Figure 8.**
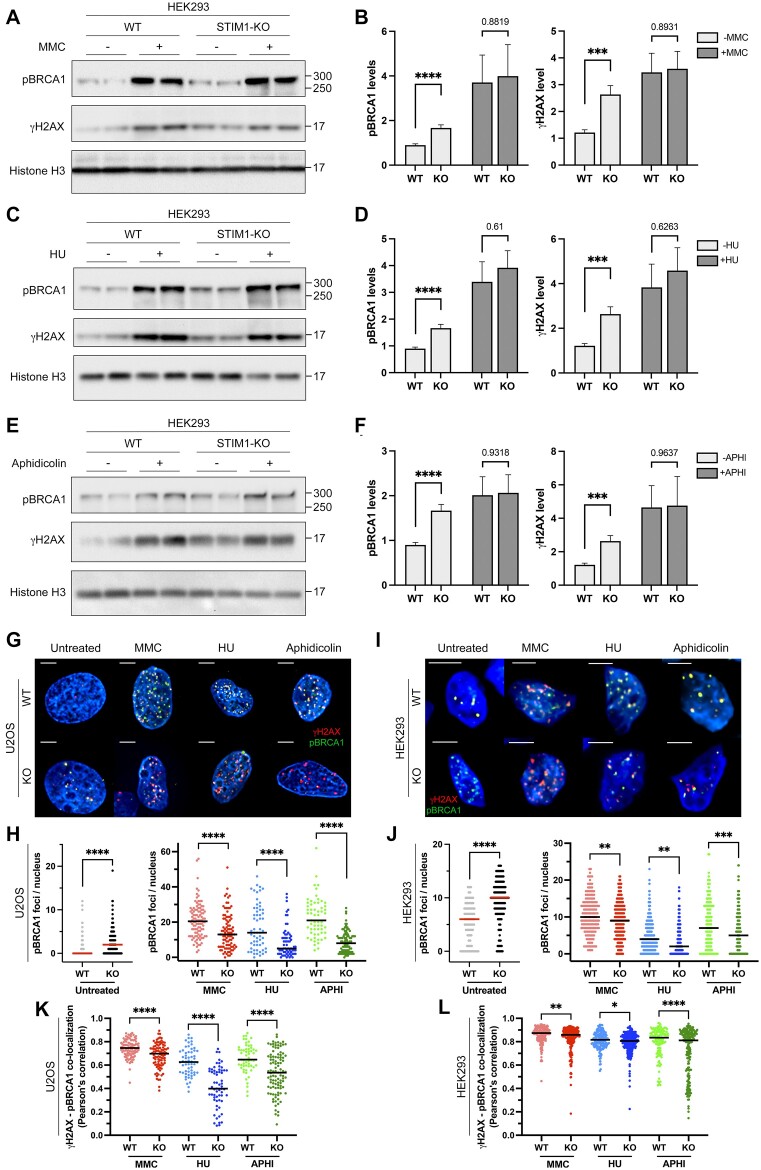
DNA damage response in the absence of STIM1. (A-F) Levels of pBRCA1 (phospho-Ser1524) and γH2AX were analyzed in nuclear lysates from HEK293 cells (wild-type and STIM1-KO). Cells were subjected to different treatments: 40 ng/ml MMC for 18 h (panels **A**, **B**), 0.2 mM HU for 24 h (panels **C**, **D**), or 0.2 μM aphidicolin for 24 h (panels **E**, **F**). Quantification of data from immunoblots is shown in panels B, D and F (from a minimum of *n* = 3 technical replicates and *n* = 2 biological samples in all cases). Quantification was carried out by calculating the volume of the pBRCA1 or the γH2AX band relative to the volume of the histone H3 band. Data were normalized to untreated wild-type cells. (**G–J**) Immunolocalization of pBRCA1 (green) and γH2AX (red) in both U2OS (panel G) and HEK293 cells (panel I), untreated or treated with MMC, HU, or aphidicolin as indicated in Materials and methods section for immunolocalization experiments. Quantification of pBRCA1 foci in U2OS cells (panel H) and HEK293 cells (panel J) from immunostaining assays shown in panels G-I. (**K**, **L**) Colocalization of pBRCA1 and γH2AX from immunostaining assays in U2OS (panel K) and HEK293 cells (panel L). U2OS cells analyzed: *n* = 121 (WT); *n* = 102 (WT + MMC); *n* = 59 (WT + HU); *n* = 59 (WT + aphi); *n* = 120 (KO); *n* = 94 (KO + MMC); *n* = 65 (KO + HU); *n* = 95 (KO + aphi), from *n* = 2 biological replicates in all cases. HEK293 cells analyzed: *n* = 254 (WT); *n* = 175 (WT + MMC); *n* = 259 (WT + HU); *n* = 153 (WT + aphi), *n* = 212 (KO); *n* = 223 (KO + MMC); *n* = 201 (KO + HU); *n* = 201 (KO + aphi), from *n* = 2 biological replicates in all cases.

To clarify the role of STIM1 in DDR pathways, we monitored the levels of pBRCA1 (phospho-Ser1524) and γH2AX (Figure 8A-F). In addition to the elevated levels of γH2AX previously shown in Figure 7, we observed that STIM1-deficient cells exhibited increased levels of pBRCA1 compared to wild-type cells in the absence of genotoxic stress. Since BRCA1 plays a crucial role in DNA repair by homologous recombination (HR), these findings suggested a close relationship between the basal DNA damage in STIM1-KO cells and this pathway, which is predominant in the S/G2/M phase.

We also monitored these two markers in cells treated with MMC (Figure 8A, B), hydroxyurea (HU, Figure 8C, D), an inducer of DSBs, or aphidicolin (Figure 8E, F), a DNA polymerase inhibitor and therefore an indirect inducer of DNA damage. In all cases, cells showed increased levels of DNA damage markers, γH2AX and pBRCA1. However, the increase in γH2AX and pBRCA1 levels were only slightly higher in KO cells upon treatment with MMC, HU or aphidicolin, when compared with the parental cell line, and these differences did not reach statistical significance as assessed by immunoblotting.

Due to the differences in the basal level of pBRCA1 in wild-type and STIM1-KO cells, we carried out an immunolocalization study to detect pBRCA1 and γH2AX foci (Figure 8G–J). Interestingly, we observed two significant findings: (i) the baseline levels of pBRCA1 foci were higher in STIM1-KO cells, both in U2OS (Figure 8G, H) and HEK293 (Figure 8I, J), corroborating our observations from immunoblotting; (ii) the levels of pBRCA1 foci significantly increased following treatment with MMC, HU or aphidicolin in both cell types. However, in STIM1-KO cells, the number of pBRCA1 foci formed was significantly lower under all experimental conditions. For this reason, we assessed the number of nuclear γH2AX foci in cells treated with HU or aphidicolin (Supplementary Figure S5). The results demonstrated that STIM1-deficient cells exhibited increased levels of DNA damage markers. Notably, the degree of colocalization between pBRCA1 and γH2AX decreased in the absence of STIM1 for both cell types and under all conditions (MMC, HU, aphidicolin) (Figure 8K, L). These data suggest that STIM1 may be necessary for the homologous recombination (HR)-dependent response to replication stress.

**Figure 9.**
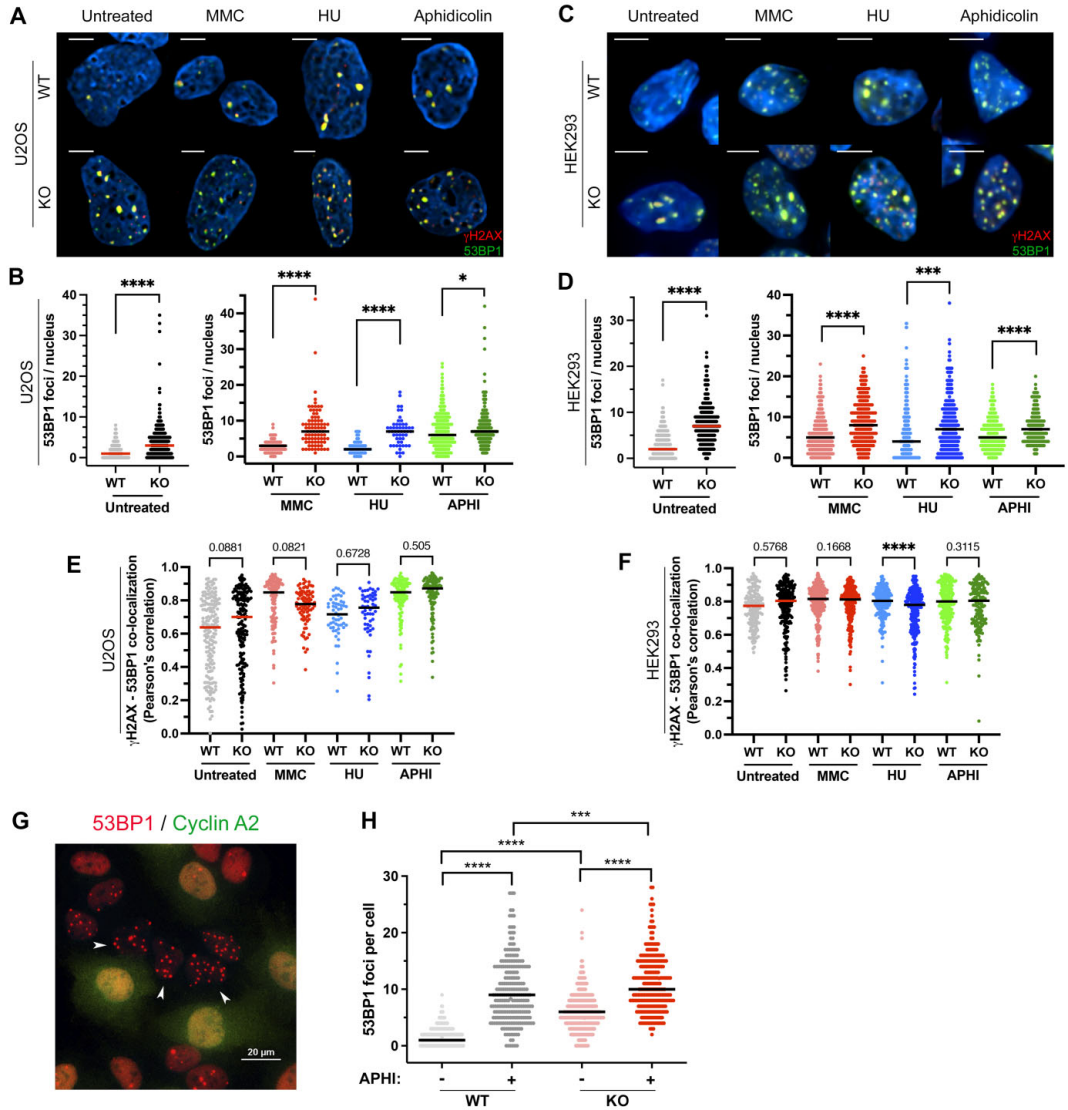
Assessment of γH2AX and 53BP1 foci. (**A–D**) Immunolocalization of 53BP1 (green) and γH2AX (red) in U2OS (panel A) and HEK293 cells (panel C), untreated or treated with MMC, HU, or aphidicolin, as indicated in Materials and methods section for immunolocalization experiments. Quantification of 53BP1 and γH2AX foci in U2OS cells (panel B) and HEK293 cells (panel D) from immunostaining assays shown in panels A, C. (**E**, **F**) Colocalization of 53BP1 and γH2AX from immunostaining assays in U2OS (panel E) and HEK293 cells (panel F). U2OS cells analyzed: *n* = 393 (WT); *n* = 118 (WT + MMC); *n* = 56 (WT + HU); *n* = 214 (WT + aphi), *n* = 309 (KO); *n* = 84 (KO + MMC); *n* = 53 (KO + HU); *n* = 218 (KO + aphi), from *n* = 2 biological replicates in all cases. HEK293 cells analyzed: *n* = 273 (WT); *n* = 330 (WT + MMC); *n* = 294 (WT + HU); *n* = 247 (WT + aphi), *n* = 232 (KO); *n* = 242 (KO + MMC); *n* = 304 (KO + HU); *n* = 154 (KO + aphi), from *n* = 2 biological replicates in all cases. (**G**) U2OS cells (WT and STIM1-KO) were treated with 0.2 μM aphidicolin for 48 h, and then stained for the immunolocalization of 53BP1 foci (red) and cyclin A2 (green). (**H**) The number of 53BP1 foci per cell in cyclin A2-negative cells is plotted from *n* = 2 biological replicates (*n* = 4 total replicates). Number of cells analyzed: WT = 606; WT + aphi = 238; KO = 393; KO + aphi = 270.

After a replication stress, the two major restart fork pathways are governed by 53BP1 and BRCA1, which are known to control the pathway choice to repair DSBs. In the absence of BRCA1, an activation of the 53BP1 pathway can be observed. Moreover, the instability of genome in BRCA1-deficient cells can be corrected by disrupting 53BP1 (53–55). We analyzed 53BP1 and γH2AX foci under the same experimental conditions as described above, for both cell types (Figure 9A–D). The results indicated that, similar to what was observed for pBRCA1, the basal levels of 53BP1 foci were higher in the absence of STIM1, reinforcing the conclusion that there is more endogenous DNA damage in the absence of STIM1. Furthermore, treatment with MMC, HU and aphidicolin increased the number of 53BP1 foci, with a more significant increase in STIM1-KO cells, whether U2OS or HEK293 (Figure 9B, D). Additionally, the colocalization between 53BP1 and γH2AX foci increased upon exposure to MMC, HU, and aphidicolin, but colocalization was not affected by the absence of STIM1, except in the case of HEK293 cells treated with HU, where the decrease of Pearson's correlation was statistically significant but minimal (from 0.79 to 0.74) (Figure 9E, F).

While monitoring 53BP1 foci, we noticed the presence of 53BP1 nuclear bodies (NBs). Following replication stress, an increase in the number of 53BP1 NBs has been observed in the subsequent G1 phase when HR is deficient (56). For this reason we exposed U2OS cells to 0.2 μM aphidicolin for 48 h, stained them for cyclin A2 to discriminate cells in G1 (negative for cyclin A2), and assessed for 53BP1 NBs formation (Figure 9G, H), as described in (57). Consistent with our analysis of γH2AX levels (Figure 7A–H) and the DNA breaks shown by the comet assay (Figure 7I), STIM1-deficient cells showed significantly higher levels of 53BP1 NBs, not only after the treatment with aphidicolin, but also in its absence (Figure 9H). This confirms the presence of replication stress associated with the endogenous DNA damage in the absence of STIM1.

### STIM1 nuclear translocation is essential for DNA protection

**Figure 10.**
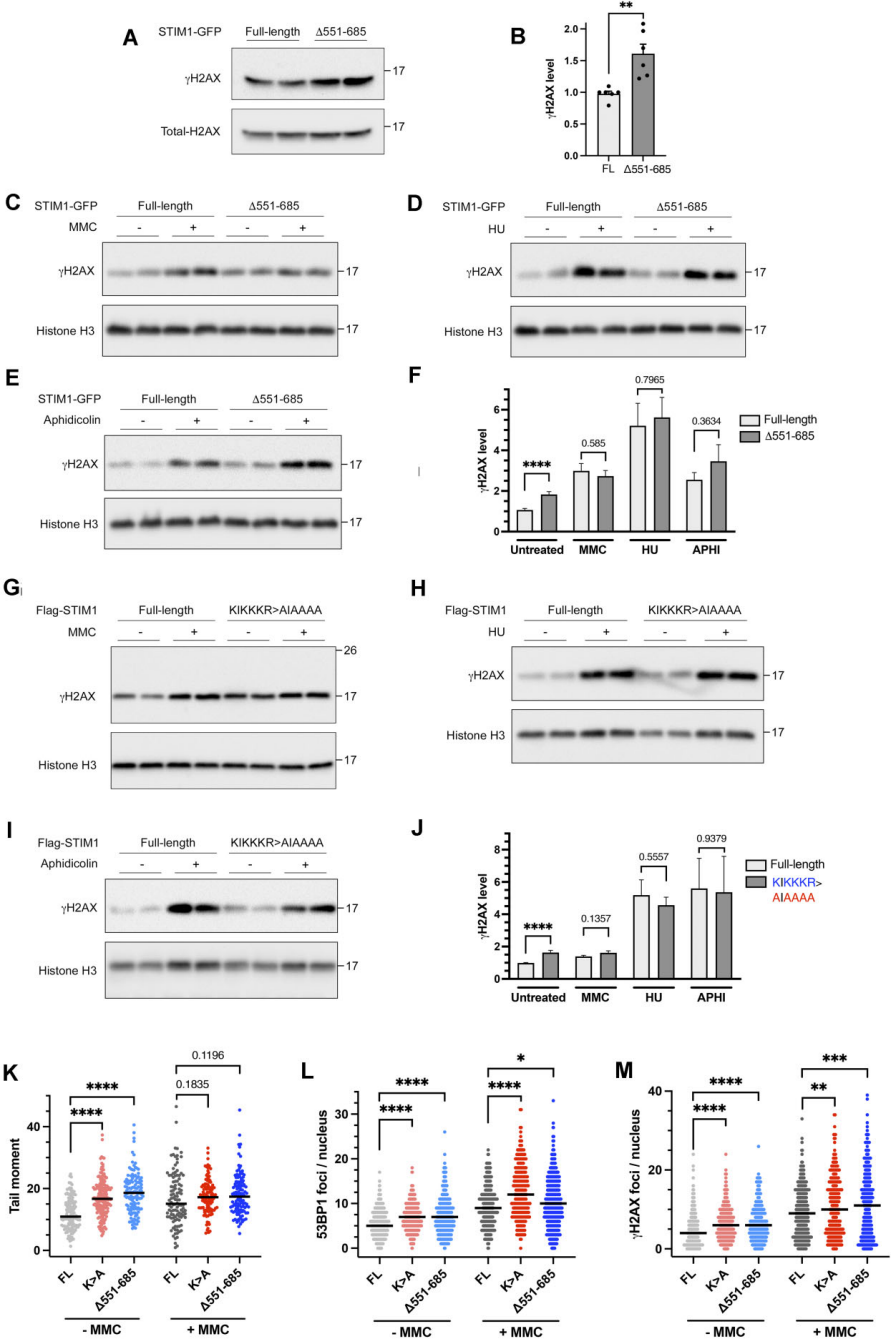
STIM1 protects cells from DNA damage. (**A**) γH2AX levels were assessed in the chromatin fraction of cells expressing full-length STIM1-GFP or STIM1(Δ551–685)-GFP. Total H2AX was assessed in parallel as a loading control. (**B**) Quantification of blots shown in panel A. Data are normalized to levels observed in HEK293 cells expressing STIM1-GFP (*n* = 3 biological samples and *n* = 6 total replicates). (**C–F**) γH2AX levels were assessed in the nuclear fraction of cells expressing full-length STIM1-GFP or STIM1(Δ551–685)-GFP treated with 40 ng/ml MMC for 18 h (panel C), 0.2 mM HU for 24 h (panel D), 0.2 μM aphidicolin for 24 h (panel E), or with the vehicle. Histone H3 was assessed as a loading control. (**F**) Quantification of blots shown in panels C–E. Data are normalized to the levels found in HEK293 cells expressing STIM1-GFP (*n* = 3 biological samples and a minimum of *n* = 4 total replicates). (**G–J**) γH2AX levels were assessed in the nuclear fraction of cells expressing Flag-STIM1 or Flag-STIM1(^382^KIKKKR^387^ > ^382^AIAAAA^387^) treated with 40 ng/ml MMC for 18 h (or with the vehicle) (panel G), 0.2 mM HU for 24 h (panel H), or 0.2 μM aphidicolin for 24 h (panel I). Histone H3 was assessed as a loading control. (**J**) Quantification of blots shown in panels G–I. Data are normalized to the levels found in HEK293 cells expressing Flag-STIM1 (*n* = 3 biological samples and a minimum of *n* = 4 total replicates). (**K**) An alkaline comet assay was carried out using STIM1-KO HEK293 cells expressing Flag-STIM1(labeled as FL), Flag-STIM1(^382^KIKKKR^387^ > ^382^AIAAAA^387^, labeled as K > A) or Flag-STIM1(Δ551–685), with or without MMC. The tail moment was quantified from the following number of cells, *n* = 130 (FL); *n* = 110 (FL + MMC); *n* = 169 (KIKKKR > AIAAAA mutant); *n* = 116 (KIKKKR > AIAAAA mutant + MMC); *n* = 131 (Δ551–685); *n* = 122 (Δ551–685 mutant + MMC), from *n* = 2 independent experiments. (**L**, **M**) Quantification of 53BP1 foci (panel L) and γH2AX foci (panel M) in STIM1-KO HEK293 cells stably and inducibly expressing Flag-STIM1 (labeled as FL), Flag-STIM1(^382^KIKKKR^387^ > ^382^AIAAAA^387^, labeled as K > A), or Flag-STIM1(Δ551–685). Number of cells analyzed for γH2AX foci assessment: *n* = 354 (FL); *n* = 357 (FL + MMC); *n* = 390 (KIKKKR > AIAAAA mutant); *n* = 288 (KIKKKR > AIAAAA mutant + MMC); *n* = 283 (Δ551–685); *n* = 344 (Δ551–685 mutant + MMC), from *n* = 2 independent experiments. Number of cells analyzed for 53BP1 foci assessment: *n* = 354 (FL); *n* = 323 (FL + MMC); *n* = 390 (KIKKKR > AIAAAA mutant); *n* = 288 (KIKKKR > AIAAAA mutant + MMC); *n* = 566 (Δ551–685); *n* = 714 (Δ551–685 mutant + MMC), from *n* = 2 independent experiments.

In previous experiments, it was demonstrated that the association of STIM1 with chromatin relies on a distal region of the C-terminal, specifically covering amino acids 551–685 (Figure 5C, D). Consequently, following the confirmation that the absence of STIM1 leads to increased DNA damage, we monitored this damage by analyzing γH2AX levels by immunoblot in cells expressing STIM1-Δ551–685, in comparison to cells expressing the full-length version of the protein (Figure 10A, B). The results revealed a significant increase in γH2AX levels in the absence of this domain.

The translocation of STIM1 to the nucleus and its subsequent enrichment in the nuclear envelope and chromatin fraction occurs in response to MMC as a model to trigger DNA damage (Figure 6E, F). Therefore, we subjected cells to MMC, HU and aphidicolin treatments in the presence of the truncated version of STIM1, STIM1-Δ551–685 (Figure 10C–F). The results showed that all DNA damage inducers increased γH2AX levels compared to the basal level. However, no significant differences were observed between wild-type and STIM1-Δ551–685 cells. Consequently, we also assessed γH2AX levels in the presence of the protein variant with a mutated NLS sequence (^382^KIKKKR^387^> ^382^AIAAAA^387^) (Figure 10G–J). In this case, a significant increase in damage in basal conditions (measured as γH2AX levels) was also observed in association with reduced nuclear translocation of STIM1 (see Figure 3D-G). Additionally, we exposed these cells to the DNA damage inducers MMC, HU and aphidicolin, but none of them increased damage in cells expressing the KIKKKR > AIAAAA variant above what was observed in cells expressing the wild-type protein.

Collectively, these results highlight the importance of STIM1 translocation to the nucleus and its association with chromatin to prevent endogenous DNA damage. However, the additional damage induced by MMC, HU or aphidicolin did not show significant alteration in the absence of STIM1 translocation, as assessed by γH2AX immunoblotting. To validate our observations, we performed a comet assay and we found that cells expressing the KIKKKR > AIAAAA variant (K > A in the figure) or the 551–685 deletion exhibited higher levels DNA breaks (Figure 10K) in the absence of DNA damage inducers. Furthermore, the analysis of nuclear foci of 53BP1 (Figure 10L) and γH2AX (Figure 10M) indicated increased levels of endogenous DNA damage when mutated STIM1 was expressed. Finally, when treated with MMC, HEK293 cells expressing the KIKKKR > AIAAAA variant or the 551–685 deletion, displayed increased levels of 53BP1 and γH2AX foci formation in response to the damage inducer when compared to parental cells (Figure 10L, M). As a control, total levels of Flag-STIM1, Flag-STIM1(KIKKKR > AIAAAA) and Flag-STIM1(Δ551–685) are shown in the Supplementary Figure S6.

### STIM1 regulates FANCD2 localization in the nucleus

**Figure 11.**
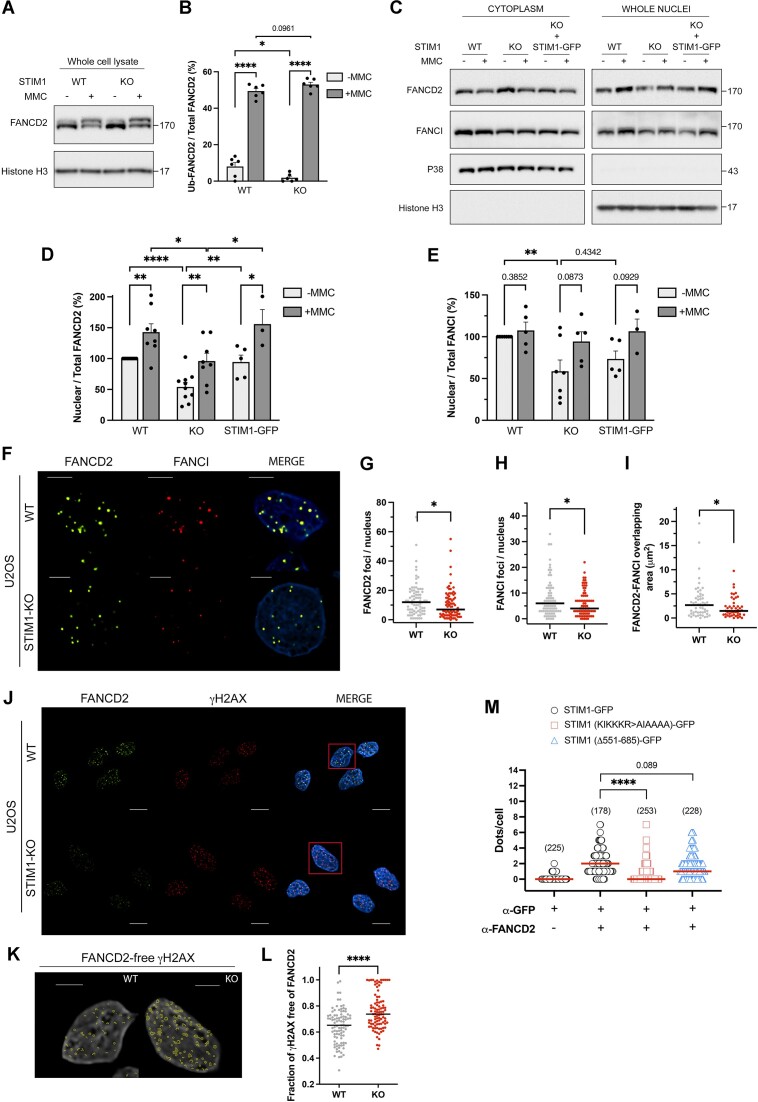
STIM1 has a role in FANCD2 association to DNA damage sites. (**A**) The levels of FANCD2 and monoubiquitylated FANCD2 were assessed by immunoblotting. Whole cell lysates (30 μg protein/lane) from HEK293 cells (WT and STIM1-KO) treated with 40 ng/ml MMC for 18 h (or with the vehicle) were prepared using RIPA lysis buffer supplemented with iodoacetamide. (**B**) Quantification of the ratio ubiquitylated FANCD2 over total FANCD2 (Ub-FANCD2 + non-Ub-FANCD2) was calculated from *n* = 6 blots (*n* = 3 biological replicates). (**C**) HEK293 cells (WT, STIM1-KO and STIM1-KO + STIM1-GFP) were treated with 40 ng/ml MMC for 18 h (or with the vehicle). Nuclear and cytoplasmic fractions were isolated, and the levels of FANCD2 and FANCI were assessed by immunoblotting (20 μg protein/lane in cytoplasm and 30 μg protein/lane in nuclear fractions). P38 MAPK and histone H3 were evaluated as cytoplasmic/nuclear marker and loading controls. (**D**, **E**) Quantification of the nuclear/total ratio (i.e., nuclear/(nuclear + cytoplasmic)) of FANCD2 (panel D) and FANCI (panel E) from blots shown in panel C (*n* ≥ 3 biological replicates and *n* ≥ 3 technical replicates in all cases). (**F**) FANCD2 (green) and FANCI (red) foci were evaluated by immunolocalization in U2OS cells treated as in panel A. Scale bar = 5 μm. (**G**, **H**) The individual number of foci per nucleus was counted in WT cells (*n* = 81) and STIM1-KO (*n* = 81) U2OS cells from two biological replicates. (**I**) The area of overlapping FANCD2 and FANCI foci was calculated from the immunolocalization experiments shown in panels F–H. (**J**) Co-localization between γH2AX foci (red) and FANCD2 foci (green) in U2OS cells treated with MMC, as indicated in panel A. Bar = 10 μm. Merged red and green channels are shown to indicate overlapping of foci. (**K**) Cells were individually analyzed to record the fraction of γH2AX without overlapping FANCD2. As an example, two cells from panel J have been selected, magnified, and binary masks created to delineate areas of FANCD2-free γH2AX foci. In this panel, the subtraction γH2AX minus FANCD2 is shown as a yellow mask. Bar = 5 μm. (**L**) Areas of the resulting subtractions from panel J are shown for WT cells (*n* = 93) and STIM1-KO U2OS cells (*n* = 95). (**M**) Proximity ligation assay to assess the interaction between FANCD2 and STIM1-GFP, STIM1(KIKKKR > AIAAAA)-GFP or STIM1(Δ551–685)-GFP. Methanol-fixed cells were incubated with the specified antibodies (rabbit anti-FANCD2 and sheep anti-GFP). The background signal with the anti-GFP antibody is shown in the first bar. Other negative controls are shown in Figure 6D. The number of red dots per nucleus is shown, and the number of analyzed cells is indicated in parentheses.

Monoubiquitylation of FANCD2 is essential for DNA binding and the subsequent monoubiquitylation of FANCI, forming the di-monoubiquitylated FANCD2/FANCI complex, which acts as a DNA clamp (58). Therefore, we monitored the levels of Ub-FANCD2 in response to MMC treatment (Figure 11A, B) and observed that the Ub-FANCD2/total FANCD2 ratio in wild-type and KO cells remained unchanged after MMC treatment. However, basal levels of Ub-FANCD2/total FANCD2 ratio (i.e. without MMC) were lower in STIM1-KO cells when compared to wild-type cells. Given the co-precipitation of STIM1 with FANCD2 shown in previous experiments, we investigated whether STIM1 might regulate the transport or access of FANCD2 to chromatin. We analyzed the nuclear/total FANCD2 ratio by immunoblot, which was significantly lower in STIM1-KO cells (Figure 11C, D). Subsequently, we assessed this ratio after MMC treatment and observed an increase in nuclear FANCD2 in both WT and STIM1-KO cells. Nevertheless, the quantity of nuclear FANCD2 remained lower in STIM1-deficient cells compared to WT cells, even after MMC treatment (Figure 11C, D). Moreover, the overexpression of STIM1-GFP in STIM1-KO cells efficiently rescued this phenotype, normalizing cytosolic and nuclear FANCD2 levels, both under basal conditions and after MMC treatment. We also examined the cytosolic and nuclear levels of FANCI (Figure 11C, E) and observed a similar pattern to that seen for FANCD2, albeit to a lesser extent. There were no significant changes in FANCI levels between the experimental samples with MMC treatment, although there was a significant decrease in nuclear FANCI levels in STIM1-KO cells in the absence of MMC.

The function of FANCD2/FANCI in replication stress is mediated by their recruitment to DNA lesions, which can be monitored by the analysis of FANCD2/FANCI foci. We performed immunofluorescence analysis in U2OS cells under MMC treatment (Figure 11F–I) and observed reduced recruitment of FANCD2 and FANCI into foci in response to MMC in STIM1-deficient cells, as well as a decreased total area of FANCD2/FANCI colocalization, indicating deficient FANCD2/FANCI complex formation. The decrease in the formation of FANCD2 foci in STIM1-deficient cells in response to MMC was also observed in HEK293 cells (Supplementary Figure S7). These observations collectively demonstrate the necessity of STIM1 for FANCD2 nuclear localization and foci formation during the repair of genotoxic-induced damage, which could account for the increased replication stress observed in STIM1-deficient cells (Figure 9G, H). This requirement is further supported by the co-localization data of γH2AX and FANCD2 in the nuclei of MMC-treated U2OS cells (Figure 11J–L). This colocalization reveals an increased level of γH2AX areas without overlapping FANCD2 in STIM1-deficient cells, indicative of more intense damage due to reduced DNA repair capacity in the absence of STIM1.

Finally, the quantification of the interaction between STIM1-GFP and FANCD2 was analyzed by PLA (Figure 11M), revealing that this interaction decreases when a variant of STIM1 with a mutated NLS (KIKKKR > AIAAAA) is expressed or when a variant lacking the chromatin interaction domain is expressed, although in the latter case, the reduction does not reach statistical significance.

In summary, the data suggest that the translocation of STIM1 into the nucleus is crucial for the optimal function of the FANCD2/FANCI-dependent signaling pathway, while the binding of STIM1 to chromatin has a more marginal effect on this specific role.

## Discussion

Our data from the STIM1 interactome analysis reveal numerous proteins related to cytoplasm-to-nucleus transport and DNA damage repair. The data shown here serve as evidence confirming these interactions and point out to a new function for STIM1 in the control of endogenous DNA damage and the repair of DNA damage caused by ICLs. Of relevance is the presence of a pool of STIM1 protein in the INM, and the fact that nuclear STIM1 levels increase in response to the generation of DNA damage by ICLs. This increase in STIM1 levels in the nucleus is mediated by a canonical NLS found in the coiled-coil domain 2 (CC2) of STIM1. This CC2, together with the CC3 domain, constitutes a domain of ∼110 amino acids responsible for the binding to the plasma membrane Ca^2+^ channel ORAI1 in response to the partial emptying of intracellular calcium stores (6,7). These two domains form a homodimeric hairpin motif (59), which is folded over the CC1 domain when the protein is in a resting state, whereas they dissociate from CC1 due to a conformational change in which the cytosolic domain of STIM1 adopts a more extended conformation upon store depletion (60). This conformational change allows the ^382^KIKKKR^387^ sequence to become more accessible to other interactors when STIM1 becomes activated. Furthermore, this lysine-rich sequence does not play a role in maintaining the closed conformation, as mutation of ^382^KIKKK^386^ to ^382^QIQQQ^386^ does not cause extension of the cytosolic domain (60). Therefore, the role for the sequence ^382^KIKKKR^387^ as NLS shown here, does not compete with other functions already described for the CC1–CC2–CC3 domains of STIM1. This conclusion aligns with the capacity of the ^382^AIAAAA^387^ mutant to rescue SOCE when overexpressed in STIM1-KO HEK293 cells (see Supplementary Figure S8). Additionally, we observed that the deletion of sequences 443–550 or 551–685 led to an increase in the nuclear steady-state STIM1 (see Figure 3B-C). Although the lack of reliable structural information for this part of the protein does not facilitate the generation of an explanation for this result, two possibilities are plausible: the necessary occurrence of nuclear export sequences that could be mapped to these sequences, or a conformational change triggered by the deletion of these sequences that exposes the ^382^KIKKKR^387^ motif, two hypothesis that are beyond the scope of this work.

The interaction with importins, determined by tandem affinity purification and mass spectrometry (MS) experiments designed to identify interactors, was already known from an initial work with STIM1 (61). However, no additional information on these interactions exists in the literature, nor has further confirmation beyond the initial MS-based description been carried out. As a result, no nuclear function has been attributed to STIM1 to date. Being part of the ER, it is logical to consider that STIM1 may be located at the outer nuclear membrane (ONM), but our experiments demonstrate that there is translocation to the INM in response to DNA damage. Furthermore, STIM1 interacts with chromatin, similar to what is observed for other proteins that cooperate with members of the DDR pathway.

Our data reveal an association between STIM1 deficiency and increased basal DNA damage in both HEK293 and U2OS cells. It is well known that such DNA damage leads to different cellular phenotypes including senescence (reviewed in (62)). In this sense, it was reported that STIM1-defective SH-SY5Y cells exhibit senescence markers, such as an upregulation of cyclin-dependent kinase inhibitor 1 (also known as p21CIP1 or p21), and increased senescence-associated beta-galactosidase activity (30). The collective evidence from this previous research together with the data shown in this work, suggests a direct or indirect link between STIM1 and physiological processes that protect cells against senescence, possibly through involvement in the DNA damage response (DDR) pathway.

A definitive strengthening for the hypothesis that STIM1 has a role on DNA repair is the finding that STIM1 co-precipitates with FANCD2-FANCI (also called ID2 complex). These two proteins form a heterodimer in response to DNA damage and associate to chromatin to activate downstream DNA repair signaling (63). There are several alternative models that account for the repair of ICLs, but all of them involve the recruitment of FANCD2-FANCI to the DNA lesion (64,65). This complex becomes ubiquitylated by a still not fully defined mechanism, although it is known that FANCD2 and FANCI monoubiquitylation are interdependent (58). Importantly, ubiquitylation of the ID2 complex enhances its binding to DNA, and specifically to ICLs [reviewed in (66)]. This monoubiquitylation is a critical step in the FA pathway as it is required for the recruitment of FAN1 (67,68) and XPF-ERCC1 (69) nucleases to the ICL. In this regard, the transport of FA proteins to the nucleus becomes critical. This transport has been proposed to be mediated by the transcription factor CEBPD and IPO4 in the case of FANCD2 (70) or in a coordinated manner for the FANCD2-FANCI complex (71). Here, we show that STIM1-defective cells exhibited reduced lower levels of nuclear FANCD2 and weakened FANCD2-dependent response to ICL, as indicated by the reduced chromatin-associated FANCD2 foci. However, this result does not necessarily imply a direct role of STIM1 in FANCD2 translocation to the nucleus, and other possibilities could explain these results. For instance, the absence of STIM1 could have an impact on the redirection of FANCD2 to DNA lesions, potentially leading to faster nuclear export and lower steady-state FACD2 levels. In support of this, lamin A/C-deficient cells have a normal response to ionizing radiation but are sensitive to agents that cause interstrand cross-links (ICLs) or replication stress because they show decreased recruitment of FANCD2 and other repair factors (72). Thus, the relocalization of DNA lesions to the nuclear envelope, and more precisely to the INM and the nuclear pore complex is supported by increasing body of evidence (73–75). Given the specific localization of nuclear STIM1 at the INM, where it accumulates in response to MMC, it is plausible to suggest a role for STIM1 in the relocalization of chromatin to the nuclear envelope. In support of this idea, the deletion of a large fragment of STIM1 that mediates association to chromatin disrupts the protective role of STIM1, as shown in this work. However, it is important to note that MMC induces not only the formation of ICLs but also monoadducts and intrastrand crosslinks (76), making necessary additional studies to set the specific type of lesions in which STIM1 is involved.

The generation of damage induced by MMC is primarily due to the replication fork stalling induced by interstrand crosslinks (ICLs). In our study, not only MMC but also HU or aphidicolin, inhibitors of DNA replication (77,78), caused increased damage in STIM1-deficient cells. These findings suggest increased replicative stress in the absence of STIM1, as evidenced by the quantification of 53BP1 NBs in G1-phase cells. Damage associated with replicative stress is predominantly repaired through homologous recombination (HR) (79). It is well established that FANCD2 forms foci with BRCA1 during the S-phase, highlighting the role of the FA/BRCA pathway in HR repair of double-strand breaks (DSBs) during this phase. Therefore, the observation that STIM1-deficient cells exhibit defects in generating pBRCA1 and FANCD2 foci points toward a deficiency in HR repair signaling. While the detailed mechanism of this defect falls outside the scope of this article and deserves further in-depth investigation, a potential possibility for future study is that this signaling is dependent on interactions with nuclear envelope scaffolding proteins, although, at present, this suggestion remains speculative.

On the other hand, our results do not support a direct role of Ca^2+^ entry through the plasma membrane Ca^2+^ channel ORAI1 on DNA repair, given that the absence of ORAI1 did not increase levels of DNA damage markers. However, STIM1 has a Ca^2+^-sensitive EF-hand domain that senses intraluminal Ca^2+^ concentration in the endoplasmic reticulum, and a drop in Ca^2+^ concentration is expected to account in the response to DNA damage, according to recent findings described by Li *et al.* (80). These authors proposed that replication stress leads to the generation of ssDNA and dsDNA that trigger the release of Ca^2+^ from the ER. Upon translocation of ssDNA/dsDNA to the cytosol, STING dissociates from the ER Ca^2+^ channel TRPV2, allowing the Ca^2+^ release from the ER and the subsequent increase of cytosolic free Ca^2+^ concentration (80). This free Ca^2+^ concentration rise stimulates AMPK-dependent phosphorylation of the nuclease EXO1, promoting its binding to 14–3–3 and inhibiting the unscheduled recruitment of the nuclease EXO1 to the replication fork (81). Although this proposal is restricted to the molecular regulation of the accessibility of EXO1 to replication forks, the initial part of the model has an unexplored consequence: the partial depletion of intraluminal Ca^2+^ concentration within the ER. It is well known that the drop of this Ca^2+^ concentration triggers an intramolecular transition of STIM1 into an open conformation, as discussed previously, leading to the exposure of coiled-coil domains CC2 and CC3 (82). This opening makes the sequence ^382^-KIKKKR-^387^, proposed here as a functional NLS, more accessible to other interactors. In this context, the translocation of ssDNA/dsDNA to the cytosol and the release of Ca^2+^ from the ER would favor the translocation of STIM1 to the nucleus, an event that is critical for an efficient repair.

The distal region of the C-terminal domain has a role in the association to chromatin, and the deletion of this domain is linked to increased DNA damage, strengthening the hypothesis that STIM1 association with chromatin is essential for efficient DNA damage repair. However, characterizing a chromatin-binding domain is hampered by the absence of structural information for this part of the protein. Notably, structural prediction with AlphaFold offers very limited confidence in predicting secondary structure elements beyond amino acid 460 (see prediction AF-Q13586-F1). Therefore, further studies are needed to uncover motifs or residues responsible for chromatin binding/association as well as their regulation by post-translational modifications, given the high number of modified sites in this part of the protein.

In conclusion, the increased level of DNA breaks in STIM1-KO cells, together with the results showing the translocation of STIM1 to the nucleus, and the fact that it regulates FANCD2 localization in the nucleus, strongly support the role of STIM1 in DNA damage repair. As a result, these findings, suggest that the potential impact of STIM1 polymorphisms should be investigated in cancer patients to learn whether these mutations play a role in this novel function. This consideration may be important in evaluating the administration of systemic agents to cancer patients with a susceptibility to DNA damage, as well as in patients with FA.

## Supplementary Material

gkae001_Supplemental_Files

## Data Availability

The data underlying this article are available in the article and in its online supplementary material. The raw files and the MaxQuant search results files have been deposited as ‘partial submission’ to the ProteomeXchange Consortium (83) via the PRIDE partner repository (84) with the dataset identifier PXD041181.
