# Correction to ‘STIM1 translocation to the nucleus protects cells from DNA damage’

**DOI:** 10.1093/nar/gkae299

**Published:** 2024-04-17

*Nucleic Acids Research*, Volume 52, Issue 5, 21 March 2024, Pages 2389–2415, https://doi.org/10.1093/nar/gkae001.

In the original publication of this article, one fluorescent micrograph has accidentally been duplicated in Figure 8I (cells treated with hydroxyurea, HU). Additionally, Figure 9A is identical to Figure 8I. The error occurred during figure assembly.

The authors have provided the original raw data, and new Figures 8 and 9.

This change does not affect the results, discussion and conclusions presented in the article.

The published article has been updated online with the new figures.

