# Supplementary material for: STIM1 translocation to the nucleus protects cells from DNA damage
Source: Nucleic Acids Res. 2024 Jan 15;52(5):2389–415. doi: 10.1093/nar/gkae001 (PMC10954485; doi:10.1093/nar/gkae001)
Supplement: gkae001_Supplemental_Files [file gkae001_supplemental_files.zip › Supplementary material corrected.pdf]

SUPPLEMENTARY TABLES AND FIGURES

STIM1 TRANSLOCATION TO THE NUCLEUS PROTECTS CELLS FROM DNA DAMAGE

by

Irene Sanchez-Lopez[1,3], Yolanda Orantos-Aguilera[1,3], Eulalia Pozo-Guisado[2,3], Alberto Alvarez-Barientos[4], Sergio Lilla[5], Sara Zanivan[5,6], Christophe Lachaud[7,8*], Francisco Javier Martin-Romero[1,3,*]

[1]Department of Biochemistry and Molecular Biology, School of Life Sciences, Universidad de Extremadura, Badajoz 06006, Spain.

[2]Department of Cell Biology, School of Medicine, Universidad de Extremadura, Badajoz 06006, Spain.

[3]Institute of Molecular Pathology Biomarkers, Universidad de Extremadura, Badajoz 06006, Spain.

[4]Bioscience Applied Techniques Facility, Universidad de Extremadura, Badajoz 06006, Spain.

[5]CRUK Scotland Institute, Switchback Road, Glasgow G61 1BD, UK.

[6]School of Cancer Sciences, University of Glasgow, Switchback Road, Glasgow G61 1QH, UK.

[7]Cancer Research Centre of Marseille, Aix-Marseille Univ, Inserm, CNRS, Institut Paoli Calmettes, CRCM, Marseille, France.

[8]OPALE Carnot Institute, Paris, France.

E-mail addresses: IS-L, isanchezlo@unex.es; YO-A, yorantosa@unex.es; EP-G, epozo@unex.es; AA-B, alalvarezb@unex.es; SL, s.lilla@beatson.gla.ac.uk; SZ, s.zanivan@beatson.gla.ac.uk; CL, christophe.lachaud@inserm.fr; FJM-R, fjmartin@unex.es

*Corresponding author: Francisco Javier Martin-Romero (Tel: +34 924 489971; e-mail: fjmartin@unex.es)

*Correspondence may also be addressed to Christophe Lachaud (Tel: +33 04 869 77381; e-mail: christophe.lachaud@inserm.fr).

Short title: STIM1 protects cells from DNA damage.

Keywords: DNA repair; Double strand breaks; Fanconi anemia; Nucleus; STIM1.

Supplementary Tables.

Supplementary table S1. List of antibodies used in the current study. The antibodies are listed together with the conditions for incubation when used for immunoblotting.

Antibody	Identifier	Source	Final concentration
Anti-STIM1	4119	ProSci, Inc	1 μg/ml in TBS-T + 10% non-fat milk
Anti-STIM1	5668	Cell Signaling Technology	5 μl antibody/2.5 mg lysate (IP only)
Anti-red fluorescent protein (RFP)	Clone 6G6	Chromotek	1 μg/ml in TBS-T + 10% non-fat milk
Anti-GFP	66002-1-Ig	Proteintech	0.3 μg/ml in TBS-T + 10% non-fat milk
Anti-IPO4	11679-1-AP	Proteintech	0.6 μg/ml in TBS-T + 10% non-fat milk
Anti-IPO7	NBP2-27150	Novus Biologicals	1 μg/ml in TBS-T + 10% non-fat milk
Anti-IPO8	sc-398854	Santa Cruz Biotechnology	0.4 μg/ml in TBS-T + 10% non-fat milk
Anti-IPO9	NB100-56499	Novus Biologicals	1 μg/ml in TBS-T + 10% non-fat milk
Anti-KPNB1	sc-136226	Santa Cruz Biotechnology	0.2 μg/ml in TBS-T + 10% non-fat milk
Anti-NUP205	sc-377047	Santa Cruz Biotechnology	0.2 μg/ml in TBS-T + 10% non-fat milk
Anti-CRM1	sc-74454	Santa Cruz Biotechnology	0.2 μg/ml in TBS-T + 10% non-fat milk
Anti-emerin	sc-25284	Santa Cruz Biotechnology	0.2 μg/ml in TBS-T + 10% non-fat milk
Anti-lamin B2	sc-377379	Santa Cruz Biotechnology	0.4 μg/ml in TBS-T + 10% non-fat milk
Anti-phospho-H2AX	9718	Cell Signaling Technology	1:4000 in TBS-T 0,1% + 5% BSA

Anti-H2AX	2595	Cell Signaling Technology	1:1000 in TBS-T 0,1% + 5% BSA
Anti-FANCD2	ab108928	Abcam	1:1000 in TBS-T + 10% non-fat milk
Anti-histone H3	17168-1-AP	Proteintech	0.1 μg/ml in TBS-T + 10% non-fat milk
Anti-p38 MAPK	9212	Cell Signaling Technology	1:1000 in TBS-T 0,1% + 5% BSA
Anti-GAPDH	sc-32233	Santa Cruz Biotechnology	0.1 μg/ml in TBS-T + 10% non-fat milk
Anti-FANCI	sc-271316	Santa Cruz Biotechnology	0.2 μg/ml in TBS-T + 10% non-fat milk
Anti-TELO2	15975-1-AP	Proteintech	0.5 μg/ml in TBS-T + 10% non-fat milk
Anti-pBRCA1	9009	Cell Signaling Technology	1:1000 in TBS-T 0,1% + 5% BSA
Anti-Flag	F1804	Merck	1:1000 in TBS-T + 3% non-fat milk
Anti-SERCA2	S1439	Merck	1:1000 in TBS-T + 10% non-fat milk
Anti-VAPB	66191-1-Ig	Proteintech	1:2000 in TBS-T + 10% non-fat milk

Supplementary table S2. STIM1 potential interactors found by mass spectrometry analysis. Hits are listed with the logFC (fold change) for the difference between samples expressing GFP and samples expressing STIM1-GFP, and the $-\log_{10}$ p-value from the t-test analysis.

Supplementary figures.

Supplementary Video 1. Localization of STIM1-GFP was assessed under super-resolution microscopy by acquiring equatorial 0.094 μm z-sections. A stack of sections with 3.4 μm in z-axis is shown. In the second half of the movie the green channel has been masked for a better visualization of the localization of STIM1-GFP.

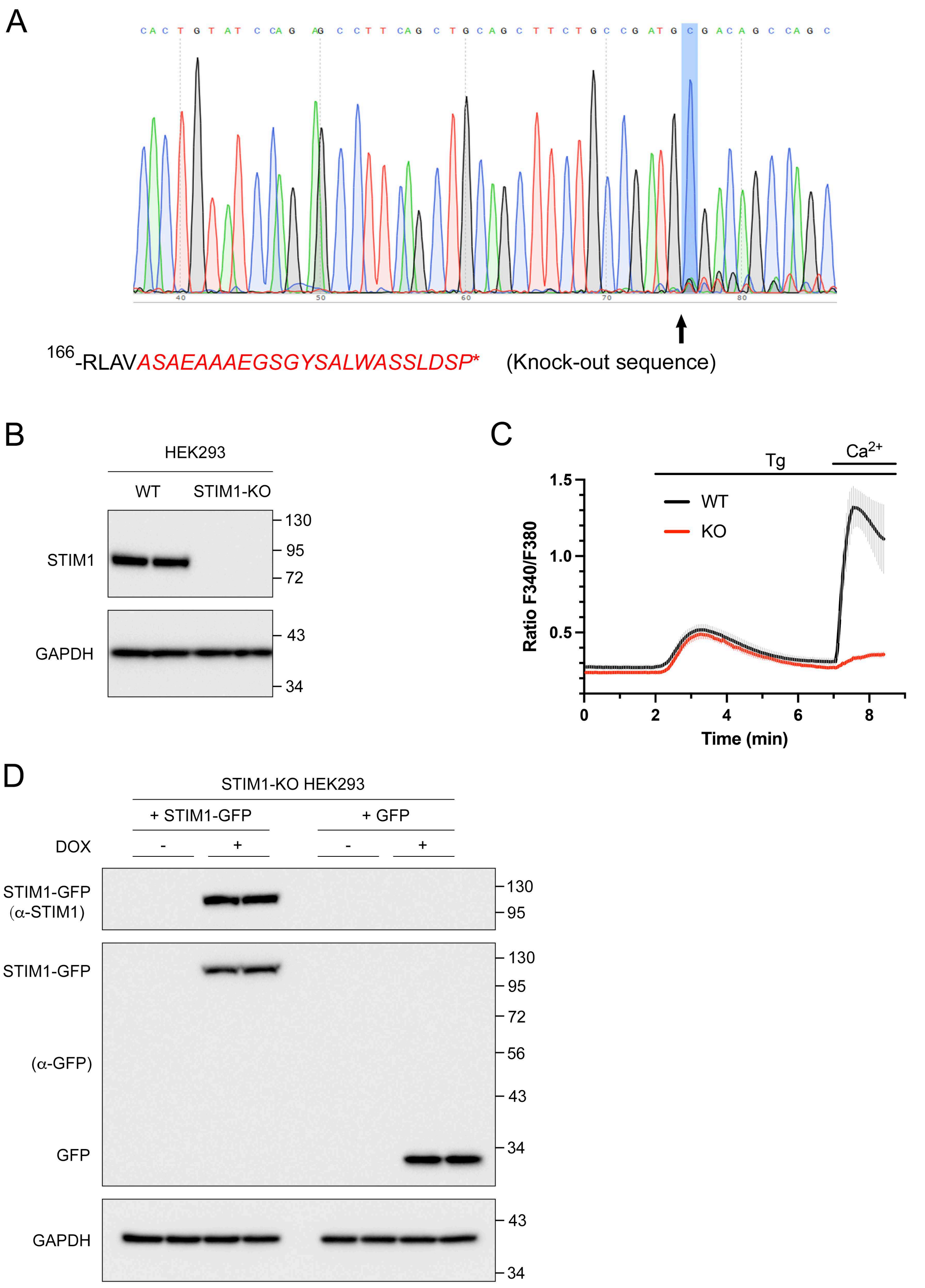

Supplementary figure 1

Supplementary figure S1. Characterization of a STIM1-knockout (KO) HEK293 cell line. HEK293 cells were subjected to CRISPR/Cas9 (D10A) editing using the strategy and the guide RNAs described elsewhere for SH-SY5Y cells (26) and U2OS cells (21). The repair of the DSB generated by Cas9 resulted in a 48-nucleotide deletion and the insertion of a single nucleotide at the target site in exon 5. (A) Chromatogram of the target site sequence using the reverse sequencing primer Seq-R: 5'-CTTTGGTTTCCATGGCACAGC. The arrow depicts the point where the indel starts, and the shaded blue base depicts the insertion. A fragment of the resulting translated protein is shown, where the new amino acids generated by the frameshift are highlighted in red italic font (the first amino acid shown corresponds to Arg166). The stop codon generated by this edition is located at exon 6 and is shown as an asterisk in the figure. (B) Immunoblotting to detect endogenous STIM1 in a wild-type HEK293 cell line, or the new STIM1-KO cell line generated in this work. GAPDH was assessed as a loading control. Twenty micrograms of protein from a whole cell lysate were used for every lane. (C) Functional characterization of the STIM1-KO cells was carried out by measuring store-operated Ca^{2+} entry. The slope of the F340/F380 ratio, monitored after Ca^{2+} addition to the assay medium, reveals the extent of SOCE, in STIM1-KO cells (red line) and wild-type cells (black line). Data are mean ± S.D. from 3 independent experiments. In the figure a single experiment has been plotted (n=12 WT cells and n=12 STIM1-KO cells). Number of cells analyzed: n=38 WT; n=35 KO cells). (D) STIM1-KO HEK293 cells stably transfected for the expression of STIM1-GFP, or GFP, were plated, and 24-36 h later, 1 μM doxycycline was added to the culture medium. After 22 h, total cell lysates were prepared to assess the doxycycline-induced expression of STIM1-GFP or GFP by immunoblot. GAPDH was assessed as a loading control.

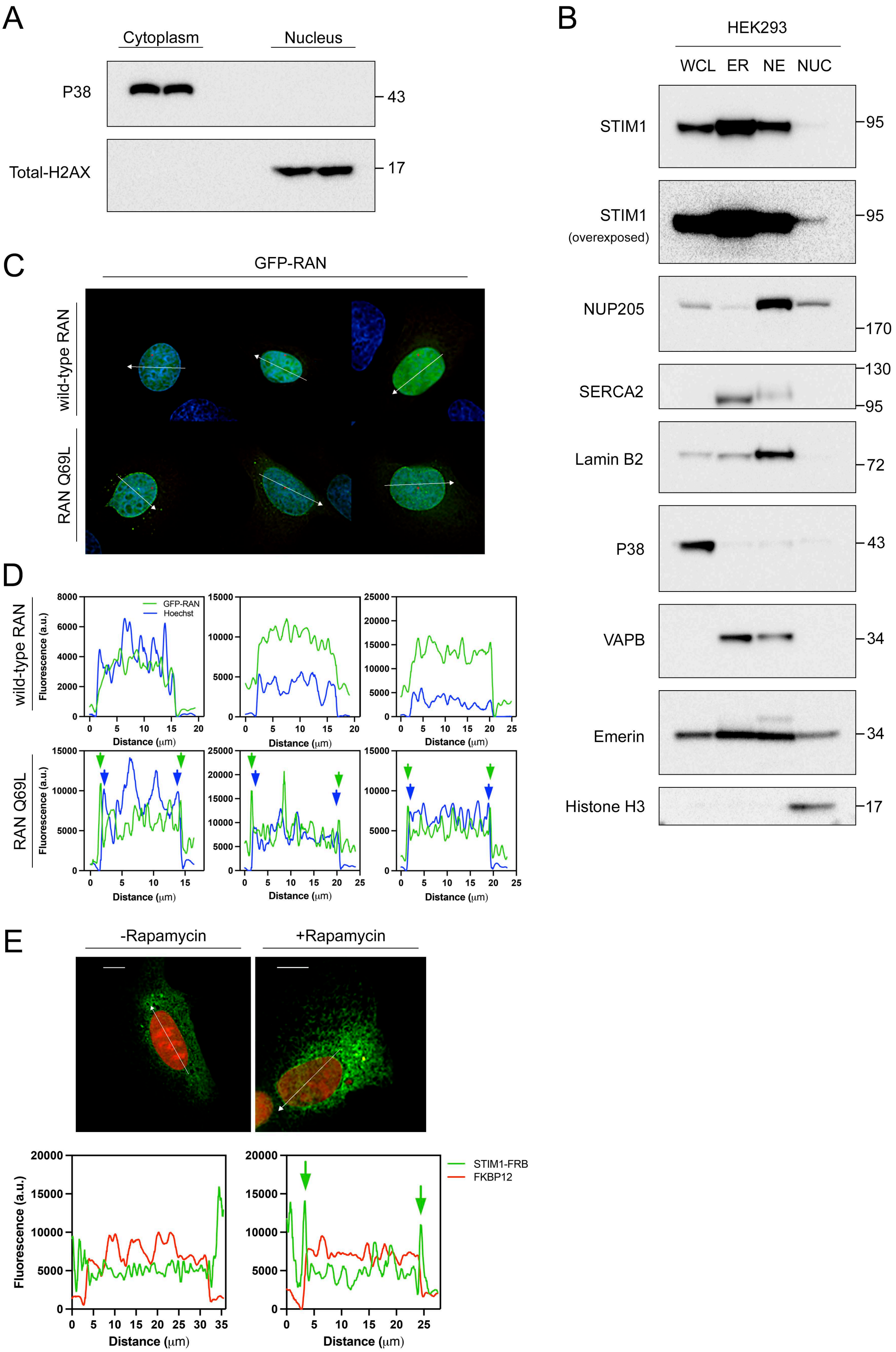

Supplementary figure 2

Supplementary figure S2. Supporting data for Figure 2. (A) After isolation of nuclear and cytoplasmic fractions, 10 μg protein/lane (cytoplasm), or 20 μg protein/lane (nucleus) were assessed by immunoblot. P38 MAPK is shown as cytoplasmic marker and loading control, whereas total H2AX was assessed as a nuclear marker. (B) Protein fractions after nuclear envelope (NE) isolation were studied in comparison with whole cell lysates (WCL), total nuclei lysates (NUC), and endoplasmic reticulum (ER). The following proteins were detected by immunoblotting in these fractions: STIM1, SERCA2 (ER-resident), VAPB (ER-resident), NUP205 (NE marker), lamin B2 (NE marker), emerin (NE marker), histone H3 (nuclear marker but absent in NE), and P38 MAPK (cytosolic marker). In all cases 15 μg protein/lane were loaded onto 4-12% acrylamide Bis-Tris gels. To enhance the visualization of STIM1 levels, the nuclear fractions is overexposed in the second blot. (C) U2OS cells constitutively expressing STIM1-mCherry were transfected for the transient expression of GFP-RAN or GFP-RAN^{Q69L}. Fixed cells were counterstained with Hoechst 33258 and observed under wide-field fluorescence microscopy. The green and blue channels are shown to illustrate the distribution of GFP-RAN. (D) The intensity of fluorescence along the arrow in panel C (intensity profile) is shown for both GFP-RAN and chromatin in an equatorial section of the nuclei. (E) U2OS cells inducibly expressing STIM1-FRB-GFP-^{2x}FRB were transfected for the transient expression of ^{3x}NLS-FKBP12-Cherry. Twenty-four hours after transfection, cells were treated with 500 nM rapamycin for 10 min (or with the vehicle) and then fixed in paraformaldehyde. The localization of Cherry- and GFP-tagged proteins was assessed in fixed cells by epifluorescence microscopy. Bar=10 μm.

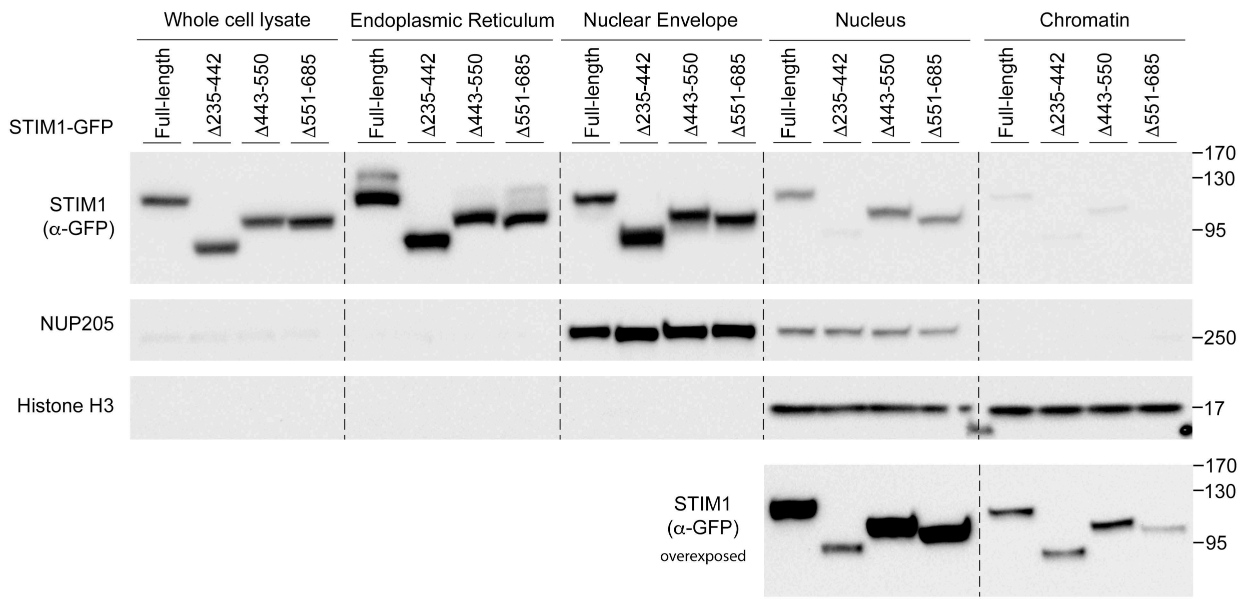

Supplementary figure 3

Supplementary figure S3. Supporting data for figure 5. Lysates were prepared from STIM1-KO HEK293 cells stably expressing full length STIM1, Δ235-442, Δ443-550, Δ551-685, all tagged with GFP. Lysates were processed to obtain whole cell lysates, ER fractions, nuclear envelope fractions, whole nuclear fractions, and chromatin fractions. In all cases, 13 μg total protein loaded. To enhance the visualization of STIM1 levels, the chromatin and nuclear fraction are overexposed in the lower panels.

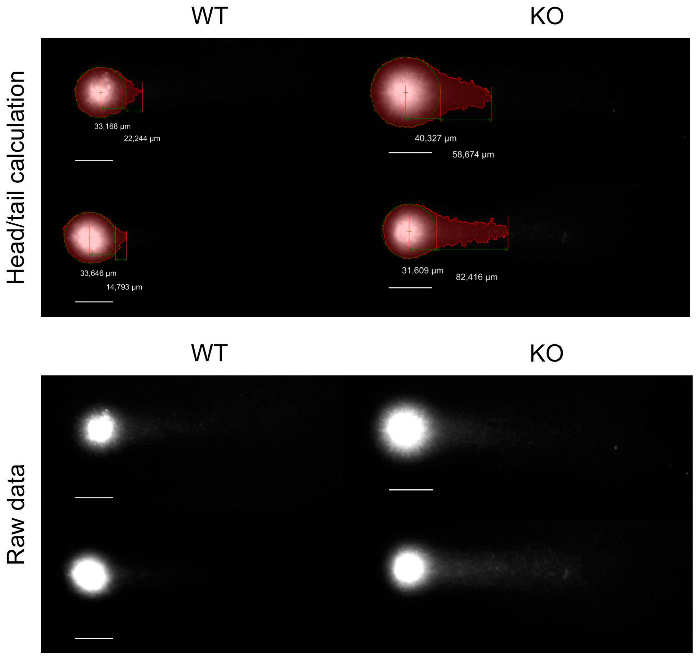

Supplementary figure 4

Supplementary figure S4. Comet assay. Representative images from comet assays performed in the parental HEK293 cell line (left) and the STIM1-KO HEK293 cell line (right). Bar=50 μm. *Top*: Panels show calculations on region of interests (ROIs: head and tail). *Bottom*: raw images used in top panels. Data acquisition and analysis were performed with the NIS-Elements AR software.

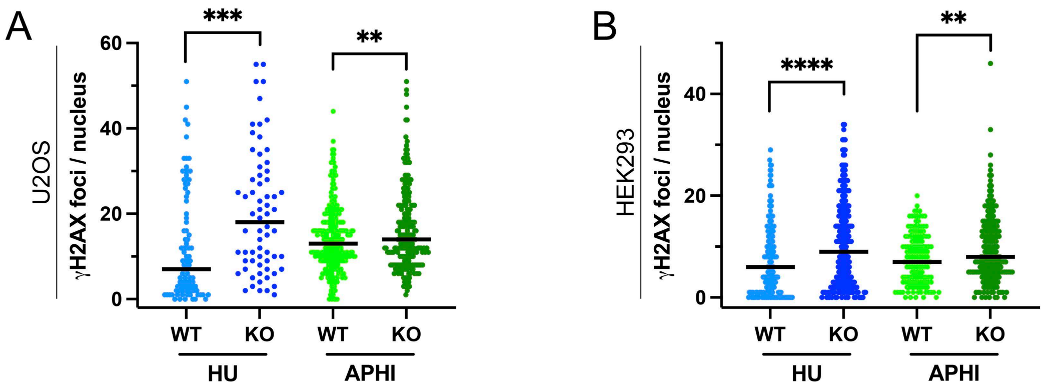

Supplementary figure 5

Supplementary figure S5. Supporting data for figure 8. Quantification of γH2AX foci in U2OS from immunostaining assays in both U2OS (panel A) and HEK293 cells (panels B), treated with HU or aphidicolin, as indicated in previous figures.

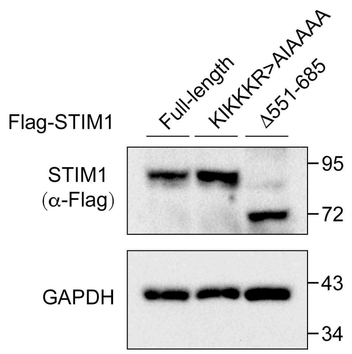

Supplementary figure 6

Supplementary figure S6. Supporting data for figure 10. Total levels of Flag-STIM1, assessed by immunoblotting in whole cell lysates from STIM1-KO HEK293 cells stably and inducibly expressing Flag-STIM1 (full length), Flag-STIM1(^{382}KIKKKR^{387}>^{382}AIAAAA^{387}), and Flag-STIM1(Δ551-685).

A

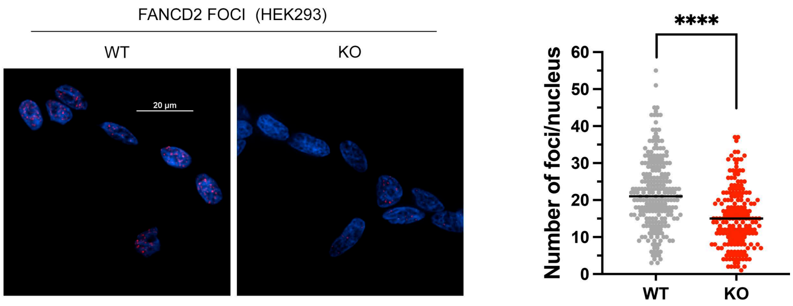

Supplementary figure 7

Supplementary figure S7. Supporting data for figure 11. Analysis of nuclear FANCD2 foci in wild-type and STIM1-KO HEK293 cells treated with 40 ng/ml MMC for 18 h. The individual number of foci per nucleus was counted in WT cells (n=242) and STIM1-KO (n=197) HEK293 cells from 4 biological replicates.

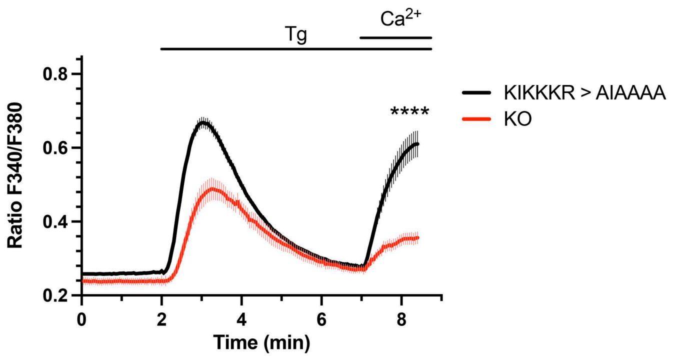

Supplementary figure 8

Supplementary figure S8. Store-operated Ca^{2+} entry assessment in cells expressing Flag-STIM1(^{382}KIKKKR^{387}>^{382}AIAAAA^{387}). STIM1-KO HEK293 cells (red line) of STIM1-KO cells expressing Flag-STIM1(^{382}KIKKKR^{387}>^{382}AIAAAA^{387}) were assessed for store-operated Ca^{2+} entry following the protocol described in the supplementary figure 1. Data are mean ± S.D. from 3 independent experiments. In the figure a single experiment has been plotted (n=12 KO cells and n=25 Flag-STIM1(K>A) cells).
